# Economic Threats, Political and National Identification Predict Affective Polarization: Longitudinal Evidence From Spain

**DOI:** 10.5334/irsp.838

**Published:** 2024-03-21

**Authors:** Efraín García-Sánchez, Felicity Turner-Zwinkels, Rebekka Kesberg, Medhi Marot, Rosa Rodríguez-Bailón, Guillermo B. Willis, Toon Kuppens

**Affiliations:** 1 Stanford SPARQ, Department of Psychology, Stanford University, CA, USA; 2 Mind, Brain, and Behavior Research Center (CIMCYC), University of Granada, Granada, Spain; 3 Department of Sociology, Tilburg University, The Netherlands; 4 Political Science Department, University of Amsterdam, The Netherlands; 5 School of Psychology, University of Sussex, United Kingdom; 6 CNRS, LAPSCO, UniversitéClermont Auvergne, Clermont-Ferrand, France; 7 Faculty of Behavioural and Social Sciences, University of Groningen, The Netherlands

**Keywords:** Affective polarization, economic threats, Economic inequality, Identity, Ideology

## Abstract

Economic threats, along with political identities and ideologies, are associated with affective polarization. However, there is still a need to learn more about the consequences of different economic threats and identities fueling polarization. We take a longitudinal perspective in testing the influence of these phenomena on affective polarization. Specifically, we tested the effect of subjective personal and collective economic threats and political, national, regional, and European identities on affective polarization towards politicians and partisans in Spain. We use four waves of the E-DEM panel study from Spain (N = 2,501) collected between 2018 and 2019. We conducted longitudinal multilevel analyses to determine the growth in affective polarization and included predictors at the between- and within-person levels. Consistent with our hypotheses, we found that collective economic threats, such as perceiving more unfairness in the distribution of wealth and being dissatisfied with the Spanish economy, positively predict affective polarization. Contrary to our expectations, personal economic threats did not predict affective polarization. Furthermore, political and national identities positively predicted affective polarization towards politicians and partisans. Interestingly, exploratory analyses suggested that the associations between economic threats, identities, and affective polarization are moderated by political ideology. We discuss how economic threats and identities may exacerbate animosities toward political actors.

Affective polarization is one of the defining topics of the current political agenda. Political events, such as the insurrection at the Capitol in the U.S. in 2021, the emergence of populist and extreme political parties in Europe, and the management of the COVID-19 crisis, have demonstrated how polarization is fed by emotions such as anger, outrage, or fear. Affective polarization—divergence of affects between groups due to different opinions around social and political issues (Lelkes, 2016)—creates a context of distrust and undermines democracy (Orhan, 2022).

However, the study of affective polarization has focused primarily on political cleavages within the United States, with particular attention to ideological differences in a two-party system (Iyengar et al., 2019), overlooking multiparty political systems and within-person changes over time (Wagner, 2021). Moreover, affective polarization has been linked to structural economic threats (Funke et al., 2016; Gidron et al., 2019), political identity (Dias & Lelkes, 2021; Iyengar et al., 2012), and ideological differences (Mason, 2018; Webster & Abramowitz, 2017), giving less attention to subjective economic threats—i.e., perceptions of potential economic harms. Subjective economic threats are crucial to understanding affective polarization because they shape cognitive-motivational processes implied in assessing in- and out-groups (Fritsche & Jugert, 2017; Klackl et al., 2022) and influence political preferences (Brandt & Bakker, 2022).

The present study examines affective polarization in Spain across four waves collected during the political crisis between 2018 and 2019 (Torcal et al., 2020). The longitudinal nature of our data allows us to account for between-person differences and within-person changes over time. This research contributes to filling some of the previously mentioned literature gaps. First, we shed light on economic threats as a predictor of affective polarization. We examine how personal and collective subjective economic threats can uniquely and independently influence affective polarization. Second, we examined the role of political, national, regional, and European identities in fueling affective polarization. In addition to political identity in predicting affective polarization (Dias & Lelkes, 2021; Iyengar et al., 2012; Torcal & Comellas, 2022; Westfall et al., 2015), national and regional identities can explain other sources of unique variance in affective polarization (Bettarelli et al., 2022). Likewise, supraordinate identities associated with European identity may imply endorsing multicultural and cosmopolitan values that likely reduce intergroup animosities (Dalton, 2021). Third, we examine affective polarization toward different political groups: politicians and partisans. Feelings toward out-group politicians were more negative than toward out-group partisans (Kingzette, 2021) or the overall opposing party (Druckman & Levendusky, 2019). Although politicians and partisans are related to politics, politicians represent political institutions. In contrast, partisans are fellow citizens, families, or friends who hardly engage actively in politics beyond voting for one party. Hence, we can examine which patterns and predictors of affective polarization are consistent across different targets (i.e., politicians and partisans) and which are specific to each political actor.

## Political and Territorial Identities on Affective Polarization

Affective polarization refers to the tendency to like or dislike people attached to political groups, such as partisans and politicians (Druckman & Levendusky, 2019; Iyengar et al., 2012). Although affective polarization can be a byproduct of ideological differences (Homola et al., 2022; Webster & Abramowitz, 2017), social identities uniquely predict affective polarization and policy preferences (Iyengar et al., 2012). From this perspective, affective polarization is fueled by partisan identity, reinforcing intergroup biases such as in-group favoritism and out-group derogation (Iyengar et al., 2019). Partisan identity increases affective polarization (Iyengar et al., 2012), guides social norms and prejudice that augment people’s feelings toward in- and out-groups (Lelkes, 2016), fosters discrimination against political counterparts (Westwood et al., 2018), and strengthens the relationship between in-party support and out-party opposition (Satherley et al., 2020). Indeed, experimental evidence suggests that political identity is a mechanism through which ideological differences influence affective polarization (Dias & Lelkes, 2021).

Apart from partisan identity, people have multiple social identities linked to the territory—e.g., city, country—and groups with shared values—e.g., pro-European Union, feminist. For instance, regional identities are positively associated with affective polarization in Spain (Rojo, 2021), and regional identities may explain why within-country variation at the regional level explains more variance in affective polarization in Europe than differences between countries (Bettarelli et al., 2022). National identity is related to less affective polarization in the U.S. because of a shared identity (Levendusky, 2018; Voelkel et al., 2022), but national identity can also be linked to nationalist and anti-immigrant sentiments that can potentially increase affective polarization toward social groups (Wojcieszak & Garrett, 2018). Similarly, European identity has conflicting implications for affective polarization: European identity is associated with values that reduce outgroup animosities (e.g., support for multiculturalism) (Dalton, 2021), but it also competes with interests coming from territorial—national and regional—and partisanship identities due to the alleged influence of Europe in a country’s internal affairs (Down & Han, 2021). Therefore, territorial identities may overlap with cultural and political values that fuel affective polarization differently, suggesting the relevance of accounting for distinct identities when studying affective polarization.

## Economic Threats and Affective Polarization

Economic threats could also account for affective polarization beyond political identities. Economic threats are defined as potential harms to people’s financial well-being that can be caused either by objective scenarios (e.g., economic inequality, unemployment, or poverty) or by subjective assessments of personal or collective situations related to the economy (Fritsche & Jugert, 2017). The literature has focused mainly on objective economic risks regarding the relationship between economic threats and affective polarization. For instance, economic crises and rising economic inequality positively relate to affective polarization toward political actors (Gidron et al., 2019; Reiljan, 2020). Similarly, economic upheavals are positively associated with greater political polarization across partisan lines in Europe (Winkler, 2019) and the U.S. (Garand, 2010). As such, objective economic threats foster uncertain conditions that could feed intergroup conflict and affective polarization.

However, the effects of objective economic threats on individuals’ outcomes depend on their subjective appraisals (Peters & Jetten, 2023; Willis et al., 2022). These subjective appraisals, also defined as subjective economic threats, refer to perceptions, assessments, or feelings that an aversive event might negatively affect their economic resources and disrupt their sense of stability and certainty (Fritsche & Jugert, 2017). Because economic threats increase uncertainty, people seek to restore control by strengthening group membership and self-stereotyping (Fritsche et al., 2011; Reiss et al., 2021), which ends up exacerbating animosities toward different social groups. For instance, subjective economic threats, such as evaluations about economic inequality, are likely to harden people’s evaluation of wealth-based groups (Jetten et al., 2021), increase a general sense of competition (Sommet & Elliot, 2023) and status anxiety (Melita et al., 2021), and reduce generalized trust and social capital (Buttrick & Oishi, 2017; García-Sánchez et al., 2024; Willis et al., 2022). These psychosocial consequences of subjective economic threats due to evaluations of inequality increase the intensity of emotional responses toward both political and non-political social groups. As such, threats attached to economic evaluations can increase affective polarization, apart from the influence of social identities related to in- and out-group partisanship (Iyengar et al., 2012). Yet, affective polarization could differ significantly depending on the target social groups.

Moreover, economic threats can be experienced at the personal or collective level. Personal economic threats are based on appraisals of people’s personal economic situation (e.g., feelings of individual relative deprivation or scarcity), and collective economic threats are based on group or societal appraisals of the economic situation (e.g., concerns about the country’s economy) (Klackl et al., 2022). The Intergroup Threat Theory supports this distinction, as concerns about physical or economic harm can be directed toward the individual—e.g., personal economic hardship, deprivation—or toward the group—e.g., the country’s economy, economic inequality (Stephan et al., 2015). Therefore, personal and collective economic threats can display different effects on people’s motivations and responses: personal economic threats mainly affect people’s sense of autonomy and personal fulfillment, while collective threats challenge people’s trust in the system and ideological beliefs that provide certainty and meaning about their environment (Reiss et al., 2021).

Evaluations of economic fairness can also be considered a subjective economic threat at the collective level because they signal potential harm to society’s economic well-being (Stephan et al., 2015). As objective economic threats refer to realistic concerns where people or situations may cause material losses and resource competition, fairness evaluations of economic distribution also indicate that something is going wrong in the economic system and should be rectified (Stephan et al., 2015). Economic fairness evaluations can also threaten people’s motivations to justify or challenge the system, influencing how people see themselves, others, and the overall system (Jost et al., 2022). Empirical research has shown that people from the political left and right identify justice-related topics related to poverty and inequality as threats that affect everyone globally (Kahn et al., 2022). Therefore, economic fairness evaluations can capture a subjective economic threat that challenges the system and guides people’s intergroup attitudes (Jost et al., 2022; Peters & Jetten, 2023).

Political ideology can also shape how subjective economic threats influence individuals’ responses. Cumulative evidence suggests that political ideology shapes how people understand and react to economic threats (Jost, 2017; Jost et al., 2003). However, further research highlights that this relationship is context-dependent, as researchers found inconsistencies across different countries and political domains (Brandt et al., 2021). Indeed, left-wing people are more concerned about global threats derived from non-intentional actions (e.g., not acting against inequality or climate change), while right-wing people are more concerned about local threats derived from intentional actions (e.g., someone committing a crime or terrorism) (Kahn et al., 2022). Therefore, economic threats can appeal to people with left- or right-wing political preferences for different motives. For instance, collective economic threats related to fairness evaluations may concern left-wing people because they are motivated to reduce inequality (Anderson & Singer, 2008), while collective economic threats related to economic losses may concern right-wing people because this indicates a system dysfunction that threatens the status quo (Jost, 2017). In both cases, threats related to economic unfairness may trigger left-wing people to support progressive social change—toward more equality and inclusion—and right-wing people to support reactionary social change—toward more inequality and exclusion (Becker, 2020). Thus, economic threats can influence affective polarization along partisan lines, yet it can be due to different motives.

## The current research

This study examines the role of subjective economic threats and different identities (i.e., partisan, regional, national, and European) on affective polarization towards politicians and partisans in Spain. We used publicly available data from a panel survey composed of four waves between the last term of 2018 and the first term of 2019 (Torcal et al., 2020). The research time frame was characterized by an acute government legitimacy crisis derived from a censure motion against the ruling government and the conflict with Catalonia over its self-proclaimed independence referendum (Torcal et al., 2020).

We test whether there was a change in affective polarization toward politicians and partisans in Spain during the six months of the study and which predictors significantly impacted it. We expect personal economic threats, such as personal economic hardship (H1a) and economic concerns (H1b), to positively predict affective polarization toward politicians and partisans. Likewise, we expect collective economic threats, such as perceived unfairness of wealth distribution (H2a) and dissatisfaction with the economy (H2b), to positively predict affective polarization toward politicians and partisans. Furthermore, identities are related to in-group favoritism and intergroup animosities. Therefore, we hypothesize that political (H3a), national (H3b), and regional (H3c) identities positively predict affective polarization toward politicians and partisans. On the contrary, we hypothesize that European identity will negatively predict affective polarization toward politicians and partisans (H3d) because supraordinate identities are related to common identities that reduce intergroup biases. It is also plausible that European identity is linked to stronger animosities toward political actors due to the emergence of Eurosceptic movements (e.g., Brexit), but empirical evidence on this relationship is scarce. All the hypotheses we have formulated (from H1a to H3d) will be tested at the between-person level since not all variables were measured in all waves. Still, we examine the within-person effects of the indicators that contain longitudinal information.

We also explore whether political ideology moderates the associations between subjective economic threats and identities and affective polarization. Yet we did not have hypotheses about specific ideological asymmetries between threats and identities on affective polarization; it is plausible that left-wing people feel more threatened by collective economic threats related to fairness evaluations because they target justice values, while right-wing populist parties may feel more threatened by collective economic threats related to economic performance because it challenges the status quo. Data and materials are available at: https://osf.io/sw8je/.

Importantly, this research was conducted in Spain, where contextual factors may also fuel affective polarization. First, Spain is among the countries in Europe with the highest levels of affective polarization (Gidron, 2019; Reiljan, 2020). Affective polarization in Spain has increased over the last decades because people moved to ideological extremes with the surge of new left and right political parties, the formation of new coalitions, territorial conflicts (e.g., the Catalonian referendum), and the legitimacy crisis of the government (Bartle et al., 2020; Torcal & Comellas, 2022). Second, territorial issues and partisanship divides are more critical in Spain for exacerbating animosities than differences between policy preferences and issue-based ideologies (Comellas & Torcal, 2023; Miller, 2020). Thus, identities linked to regions can uniquely influence affective polarization in Spain beyond partisan identity.

## Method

### Participants

We used data from the E-DEM dataset, a four-wave online panel survey conducted over a six-month period (from October 2018 to May 2019) in Spain. Because the waves were not equally spaced, we transformed this variable in elapsed months (i.e., 0, 3.23, 5.33, and 6.03 months after the first data collection) to facilitate interpretation. The effect of time on our outcome variables now indicates the expected change for each month of the study. Data was collected by a private survey company using a non-probabilistic online panel, using sample weights in terms of region or residence, gender, and age to represent the composition of the general population. From the 2,501 participants that composed the first wave, 1,484 (59.3%) remained in the four waves that included the items relevant to this study (male = 47.51%, female = 52.49%; *M_age_* = 43.38 years, *SD* = 13.73; additional sociodemographic information is available at Table S1 in the supplementary material). The sample characteristics were close to the official records of the National Institute of Statistics of Spain^1^ (see Torcal et al., 2020). The attrition analyses showed no substantial biases in the sample composition as a function of sociodemographic and focal variables of interest (reported in the supplementary material, section 2). The data is publicly available at https://data.mendeley.com/datasets/6bt6r8cn2r/3.

### Outcome variables

#### Affective polarization

We used the measure proposed by Wagner (2021), defined as an individual-level variable that accounts for the spread of like-dislike scores that each person has about different political actors (e.g., politicians, parties, etc.). More formally, it is operationalized as: \[
\sqrt {{\sum_{p = 1}^{P} {{(Like{._{ip}} -\,\underline {Like{._i}})}^2}} \over {{n_p}}}
\]. As such, *p* indicates the party, *i* is the respondent, and *like_ip_* the score given to each party p by each respondent *i*. Higher values of affective polarization indicate that people have more extreme feelings (both favorable and unfavorable) toward other political actors. We rescaled the raw scores to range between 0 and 1 to facilitate the interpretation of our results.^2^ This rescaling method allows us to interpret the regression coefficients as the expected change in the outcome variable in a proportion of its own range (not absolute values), facilitating comparisons between outcome variables. Therefore, a regression coefficient *b = 0.1* indicates that a one-unit change in the predictor is associated with a 10% increase in the outcome variable’s full range, regardless of their raw values. This transformation facilitates comparing coefficients for two outcome variables, given that the raw value of the outcome variable’s range for affective polarization toward politicians may be wider than toward partisans, hindering substantive interpretations and comparability between our tested models. This transformation was applied to both measures of affective polarization.

Affective polarization toward partisans was measured by using the feelings thermometer (ranging from 0 ‘unfavorable feelings’ to 100 ‘favorable feelings’) toward voters aligned to four political parties that represent the traditional right (PP) and left (PSOE), and the emergent right (Cs) and left (UP).^3^ The size of political parties matters for affective polarization because larger parties trigger more competition and threat than smaller parties (Wagner, 2021). Therefore, we weighted the scores according to the proportion of parliamentarians belonging to each political party that composed the Spanish parliament when the data was collected, which corresponded to the XII legislature that covered from May 19, 2016 to March 5, 2019.^4^ We compared the weighted and unweighted affective polarization for robustness checks, which did not show substantial differences in the results (see supplementary material, Section 4).

As for affective polarization toward politicians, people were asked about their feelings toward four political leaders: Pablo Casado (PP), Pedro Sánchez (PSOE), Albert Rivera (Ciudadanos), Pablo Iglesias (Unidas Podemos). We selected these four political leaders to match the political parties referred to in affective polarization toward voters. For robustness check, we also computed an alternative measure of affective polarization of politicians, adding three political leaders with regional agendas (see supplementary material, section 4).

### Predictors

All predictor variables were measured in the four waves, unless otherwise noted.

#### Personal economic concerns

Indicate participants’ self-reported economic difficulties. People were asked whether they feel concerned about a) paying their household bills, b) having to reduce their standard of living, c) having a job, d) paying off loans from the bank or paying mortgage bills (response scale ranging from 0 ‘not at all concerned’ to 3 ‘very concerned’). Average scores were computed per wave (*α_waves1,2,3,4_* = [.80, .81, .78, .81] ; *r_between-waves_* = .74).

#### Personal economic hardship

It evaluates the difficulties that participants face with their current household income. Participants should indicate which statement best describes how they feel about their current income: 1) ‘With our current income we live comfortably’, 2) ‘With our current income we get by’, 3) ‘With our current income we have difficulties’, and 4) ‘With our current income we have many difficulties’. We recoded the scale for ranging from 0 to 3 (*r_between-waves_* = .74).

#### Perceived unfairness of wealth distribution

A single item regarding participants’ evaluation of the fairness of the wealth distribution was used (‘Would you say that income and wealth are distributed fairly among regular people in Spain or that wealth should be redistributed more fairly?’). The response scale ranged from 0) ‘Wealth is fairly distributed’ to 10) ‘Wealth should be redistributed more fairly’. Higher values indicate unfair views of wealth distribution (*r_between-waves_* = .51).

#### Satisfaction with the Spanish economy

It is a single indicator of participants’ satisfaction with the Spanish economy (‘To what extent are you satisfied with the general economic situation in Spain?’). The response scale ranged from 0) ‘Completely dissatisfied’ to 10) ‘Completely satisfied’ (*r_between-waves_* = .60).

#### Territorial and supraordinate identities

It is related to the identification of groups attached to particular territories or communities (i.e., ‘We all feel more or less connected to the territory or political community (town, city, region, etc.) in which we live, but some of us feel more connected to some places than others. To what extent do you identify with the following territories?’). The territories were ‘Region or autonomous community’ (*Regional identity*) (*r_between-waves_* = .65), Spain (*national identity*) (*r_between-waves_* = .78), and Europe (*European identity*) (*r_between-waves_* = .64). The response scale ranged from 0) ‘Do not identify at all’ to 10) ‘Identify strongly’. Because these identities were asked in waves 1 and 2, we did not have enough data points (three or more) to account for within-person variance (Hoffman & Walters, 2022). Therefore, we computed the mean score of the two waves and treated it as a between-person variable.

#### Political identity

We used the participants’ perceived closeness to any political party as a proxy indicator for political identity (‘Do you consider yourself close to any political party?’). This item employed a Yes (1) or No (0) response scale (*rho_between-waves_* = .83). For robustness checks, we used an alternative measure of political identification consistent with the degree of identification with the in-group political party and found no substantial differences (see supplementary material, Table S3).

### Covariates

We controlled for sociodemographic variables: sex (0 ‘Male’, 1 ‘Female’), age (in years), education (scale ranging from 1 ‘without education’ to 8 ‘doctorate’), household income (from 1 ‘780€ or less’ to 10 ‘More than 3701€’), and area of residence (0 ‘urban’, 1 ‘rural’). We also controlled for covariates relevant to influencing affective polarization, such as political ideology (a scale ranging from 0) ‘Left’ to 10) ‘Right’); political interest as a proxy of political sophistication (‘How much are you interested in politics?’ on a scale from 1) ‘A lot’ to 4) ‘Not at all’, reverse scoring such that higher values indicate higher political interest); and using social media for discussing political issues (‘How often do you discuss politics or current political issues on social networks, Facebook, Twitter, or any other blog?’, scale from 0) ‘Never’ to 6) ‘Every day’). Furthermore, given the data collected from the 17 autonomous communities of Spain, we controlled for the gross income and economic inequality in each region.

### Procedure and analytical strategy

We conducted multilevel regression analysis for longitudinal data, modeling random intercepts for individuals and regions and using a restricted maximum likelihood estimator as suggested in the literature (Hoffman & Walters, 2022) to account for the clustered nature of the data (responses clustered within individuals and individuals nested within regions). First, we computed an unconditional growth model that included time (in months as the only predictor) to describe the linear pattern of change in affective polarization. Second, we added predictors related to economic threats at the within- and between-person levels. Third, we included identity variables. Fourth, we added contextual-level variables to examine whether socioeconomic conditions in the participants’ region of residence (i.e., Comunidad Autónoma) were linked to affective polarization. Finally, for exploratory purposes, we tested whether political ideology moderated the effect of economic threats and identities on affective polarization.

The three-level structure of our data allows us to separate within-person variation from between-person differences and between-region variability. Within-person variables were mean-centered, so the average score per participant was subtracted from each participant’s response at each wave. Between-person variables were assigned the average score of their responses across waves. Variables at the regional level were grand-mean centered by subtracting the mean value of the 17 regions from each region score. All models converged adequately and met the underlying statistical assumptions for multilevel regression models (see Section 5 in the supplementary material).

## Results

### Preliminary results

**Figure 1:**
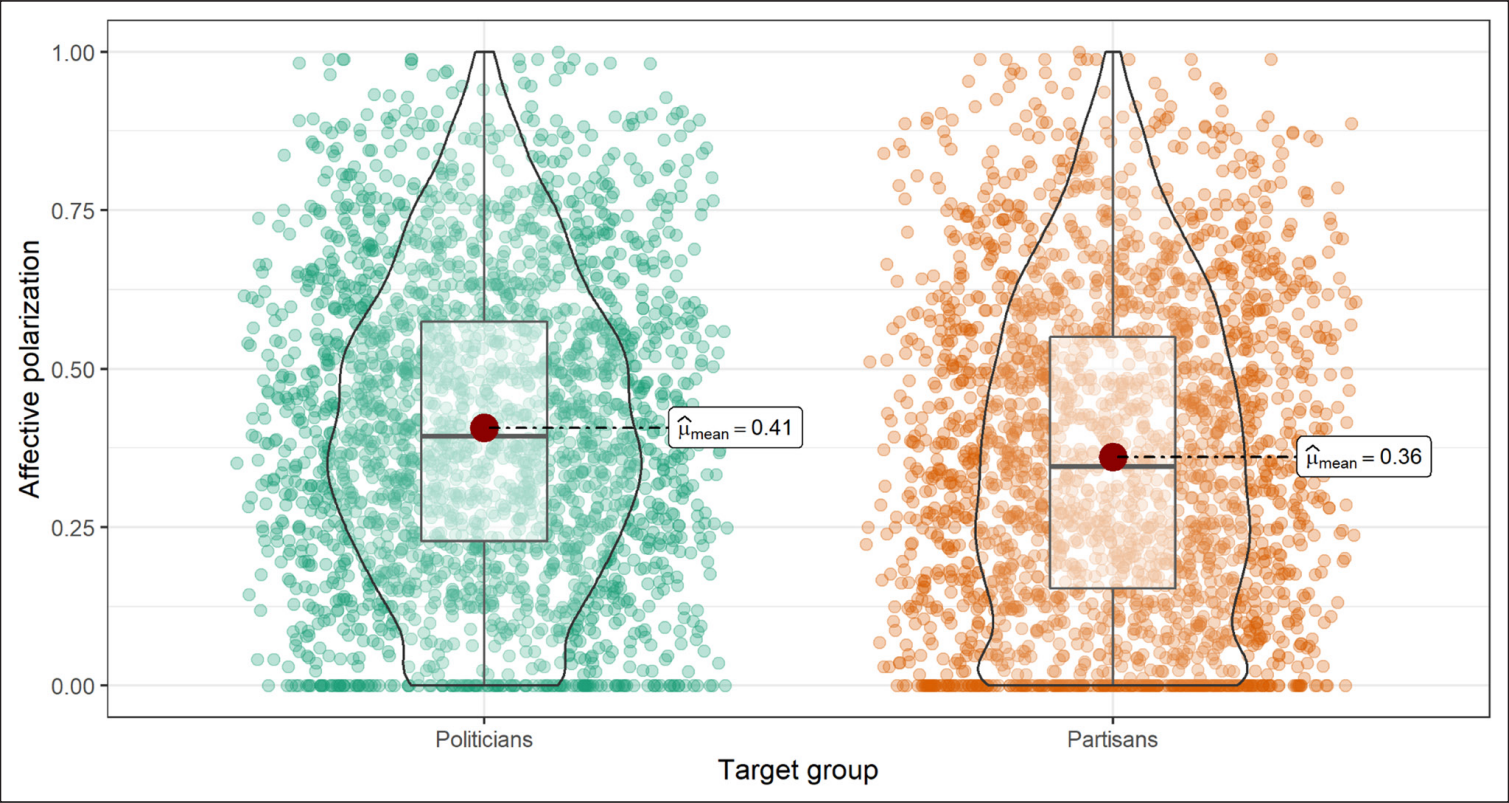
Mean (red dot), median, and interquartile range for affective polarization toward politicians and partisans.

We found different levels of affective polarization depending on the group target. On average, participants scored higher in affective polarization toward politicians (*M* = 0.41, *SD* = 0.23) than toward partisans across all waves (*M* = 0.36, *SD* = 0.25), *t(2496)* = 14.4, *95% CI* = [.04, .05], *p <* .001, *d* = 0.29 (see Figure 1). The summary statistics of the pooled sample and the Pearson correlations are reported in Table 1 (see Section 1 in supplementary material for descriptive statistics per wave).

**Table 1:** Descriptive statistics and Pearson correlations between focal variables.


	1	2	3	4	5	6	7	8	9	10	11

*1. Affective polarization toward politicians*											

*2. Affective polarization toward partisans*	.788***										

*3. Personal economic concerns*	–.073***	–.023									

*4. Personal economic hardship*	–.056**	–.012	.603***								

*5. Satisfaction with Spanish economy*	.066**	–.005	–.278***	–.312***							

*6. Perceived unfairness of wealth distribution*	.084***	.133***	.122***	.196***	–.203***						

*7. Regional identity*	.125***	.106***	–.056**	–.041*	.064**	.096***					

*8. National identity*	.072***	–.034	–.016	–.041*	.193***	–.180***	.291***				

*9. European identity*	.103***	.024	–.135***	–.157***	.274***	–.129***	.382***	.495***			

*10. Partisan identity*	.504***	.487***	–.014	0	.101***	.067***	.147***	.002	.099***		

*11. Political ideology*	–.145***	–.211***	–.036	–.074***	.082***	–.415***	–.021	.369***	.165***	–.126***	

*Mean*	0.41	0.36	1.36	2.01	3.6	8	7.32	6.78	6.47	0.5	4.02

*SD*	0.24	0.25	0.73	0.75	1.94	2.11	2.46	2.95	2.44	0.43	2.35

*Range*	0–1	0–1	0–3	1–4	0–10	0–10	0–10	0–10	0–10	0–1	0–10


*Note*: *** p < .001, ** p < .01, * p < .05; Pearson correlations with covariates are included in the supplemental material, Section 1.

### Change in affective polarization over time

The unconditional mean (intercept only) model for affective polarization toward politicians indicates that 67% of the variability is due to differences between individuals (*ICC_Level 2_ =* .67), while 66% of the variance in affective polarization toward partisans was due to differences between individuals (*ICC_Level 2_ =* .66).

Regarding the pattern of change over time, we found that time positively predicts affective polarization toward politicians (*β* = .014, SE = .007, *p* = .034) and toward partisans (*β* = .014, *SE* = .007, *p* = .047) (see Figure S1 in the supplementary material).

### Economic threats and affective polarization

Regarding the role of personal economic threats on affective polarization, we found that economic hardship and personal economic concerns at the between-person level did not predict affective polarization toward politicians or toward partisans (not supporting H1a and H1b) (see Table 2). This pattern of results was mirrored at the within-person level, with one exception: an increase in experiencing economic hardship at the within-person level positively predicted affective polarization toward partisans (but not toward politicians).

**Table 2:** Multilevel growth model for affective polarization toward politicians (left) and partisans (right).


	AFFECTIVE POLARIZATION TOWARD POLITICIANS				AFFECTIVE POLARIZATION TOWARD PARTISANS			
	
*PREDICTORS*	β *(SE)*	β *(SE)*	β *(SE)*	β *(SE)*	β *(SE)*	β *(SE)*	β*(SE)*	β *(SE)*

Time (months)	0.013	0.014	0.009	0.009	0.015*	0.016*	0.009	0.009

	(0.007)	(0.007)	(0.007)	(0.007)	**(0.007)**	**(0.007)**	(0.007)	(0.007)

*Covariates*								

Age	0.130***	0.132***	0.091***	0.089***	0.069***	0.066***	0.033	0.032

	**(0.019)**	**(0.020)**	**(0.018)**	**(0.018)**	**(0.019)**	**(0.020)**	(0.018)	(0.019)

Sex (Female)	0.025	0.025	–0.018	–0.020	0.042*	0.037*	–0.003	–0.004

	(0.018)	(0.019)	(0.017)	(0.017)	**(0.019)**	**(0.019)**	(0.018)	(0.018)

Education, 8 levels	–0.041*	–0.044*	–0.018	–0.018	–0.055**	–0.054**	–0.033	–0.032

	**(0.019)**	**(0.019)**	(0.018)	(0.018)	**(0.019)**	**(0.020)**	(0.018)	(0.018)

Income	0.057**	0.050*	0.049**	0.046*	0.021	0.023	0.020	0.020

	**(0.019)**	**(0.020)**	**(0.019)**	**(0.019)**	(0.019)	(0.021)	(0.019)	(0.019)

Political ideology (left-right)	–0.098***	–0.082***	–0.095***	–0.094***	–0.158***	–0.128***	–0.117***	–0.117***

	**(0.018)**	**(0.020)**	**(0.020)**	**(0.020)**	**(0.018)**	**(0.020)**	**(0.020)**	**(0.020)**

Rural (vs. urban)	0.010	0.011	0.015	0.015	0.013	0.011	0.014	0.012

	(0.018)	(0.018)	(0.016)	(0.016)	(0.018)	(0.018)	(0.016)	(0.016)

Media consumption (between-person)	0.003	0.006	–0.005	–0.004	0.059**	0.061**	0.043*	0.044*

	(0.019)	(0.019)	(0.018)	(0.018)	**(0.019)**	**(0.019)**	**(0.018)**	**(0.018)**

Media consumption (within-person)	0.021**	0.021**	0.021**	0.021**	0.010	0.010	0.009	0.009

	**(0.007)**	**(0.007)**	**(0.007)**	**(0.007)**	(0.007)	(0.007)	(0.007)	(0.007)

Political interest (between-person)	0.324***	0.324***	0.161***	0.162***	0.267***	0.274***	0.113***	0.114***

	**(0.020)**	**(0.020)**	**(0.020)**	**(0.020)**	**(0.020)**	**(0.020)**	**(0.021)**	**(0.021)**

Political interest (within-person)	0.025***	0.024***	0.021**	0.021**	0.031***	0.031***	0.027***	0.027***

	**(0.007)**	**(0.007)**	**(0.007)**	**(0.007)**	**(0.007)**	**(0.007)**	**(0.007)**	**(0.007)**

*Economic Threats (within-person)*								

Personal economic hardship		0.008	0.008	0.008		0.017*	0.017*	0.017*

		(0.007)	(0.007)	(0.007)		**(0.007)**	**(0.007)**	**(0.007)**

Personal economic concerns		0.001	–0.001	–0.001		0.005	0.004	0.004

		(0.007)	(0.007)	(0.007)		(0.007)	(0.007)	(0.007)

Satisfaction with economy		0.005	0.003	0.003		–0.001	–0.003	–0.003

		(0.007)	(0.007)	(0.007)		(0.007)	(0.007)	(0.007)

Perceived unfairness of wealth redistribution		0.021**	0.021**	0.021**		0.015*	0.015*	0.015*

		**(0.007)**	**(0.007)**	**(0.007)**		**(0.007)**	**(0.007)**	**(0.007)**

*Economic Threats (between-person)*								

Personal economic hardship		–0.033	–0.037	–0.037		–0.019	–0.025	–0.025

		(0.025)	(0.022)	(0.022)		(0.025)	(0.023)	(0.023)

Personal economic concerns		0.005	–0.005	–0.005		–0.011	–0.016	–0.016

		(0.023)	(0.021)	(0.021)		(0.024)	(0.022)	(0.022)

Satisfaction with economy		–0.002	–0.042*	–0.042*		–0.044*	–0.068***	–0.069***

		(0.019)	**(0.018)**	**(0.018)**		**(0.020)**	**(0.019)**	**(0.019)**

Perceived unfairness of wealth redistribution		0.037	0.019	0.019		0.058**	0.039*	0.038*

		(0.020)	(0.019)	(0.019)		**(0.021)**	**(0.019)**	**(0.019)**

*Identity (within-person)*								

Political identity			0.033***	0.033***			0.040***	0.040***

			**(0.007)**	**(0.007)**			**(0.007)**	**(0.007)**

*Identity (between-person)*								

Political identity			0.364***	0.363***			0.363***	0.363***

			**(0.018)**	**(0.018)**			**(0.019)**	**(0.019)**

Regional identity			–0.003	–0.003			0.009	0.009

			(0.018)	(0.018)			(0.018)	(0.018)

National identity			0.111***	0.117***			0.053*	0.057**

			**(0.020)**	**(0.021)**			**(0.021)**	**(0.021)**

European identity			–0.023	–0.025			–0.034	–0.035

			(0.020)	(0.020)			(0.020)	(0.020)

Economic inequality				–0.011				–0.024

				(0.016)				(0.016)

Mean income per capita				0.018				0.006

				(0.017)				(0.017)

**Random Effects**								

σ2 (within-person variance)	0.023	0.023	0.023	0.023	0.027	0.027	0.027	0.027

τ_00_ (between-person variance)	0.037	0.037	0.029	0.029	0.043	0.043	0.035	0.035

ICC	0.618	0.617	0.561	0.561	0.385	0.385	0.435	0.435

N (observations)	6951	6946	6933	6933	6962	6957	6944	6944

N (individuals)	2054	2054	2051	2051	2050	2050	2047	2047

Marginal R^2^	0.152+	0.154	0.449	0.449	0.271	0.280	0.411	0.412


*Note*: * p < .05 ** p < .01 *** p < .001; ^+^R2 estimated with no random variance at the regional level.

Concerning collective economic threats, we found that perceived unfairness in the wealth distribution (at the between-person level) positively predicted affective polarization toward partisans but not toward politicians (partially supporting H2a). This association at the between-person level means that, on average, individuals who perceived more (vs. less) unfairness in wealth distribution reported greater affective polarization toward partisans. At the within-person level, an increase in the perceived unfairness of the wealth distribution over time predicted more affective polarization toward politicians and partisans.

Furthermore, satisfaction with the Spanish economy at the between-person level negatively predicts affective polarization toward politicians (after controlling for identity variables) and partisans (supporting H2b). So, individuals who were less satisfied with the Spanish economy than the average participant showed more affective polarization. These results, however, did not hold at the within-person level.

### Identities and affective polarization

Our results suggest that political identity positively predicted affective polarization toward politicians and partisans (supporting H3a). This result was also confirmed at the within-person level, such that a higher identification with any political party over time was linked to greater affective polarization toward politicians and partisans. Robustness checks replicated these results (i.e., using an alternative indicator of political identity that accounted for the degree of closeness to their preferred political party) (see supplementary material, Table S3).

We also found that national identification positively predicted affective polarization toward politicians and partisans (supporting H3b). Although regional and European identities were positively correlated with affective polarization toward politicians (see Table 1), such associations became non-statistically significant after controlling for other variables included in the model (disconfirming H3c). Similarly, regional identity did not predict affective polarization toward partisans after controlling for covariates (disconfirming H3d) (see supplementary material, section 8, for the tests per identity variable).

Notably, region-level variables such as economic inequality and mean income per capita per region did not show statistically significant relationships with affective polarization toward politicians and partisans.

### Exploratory analyses: The moderating role of political ideology

**Figure 2:**
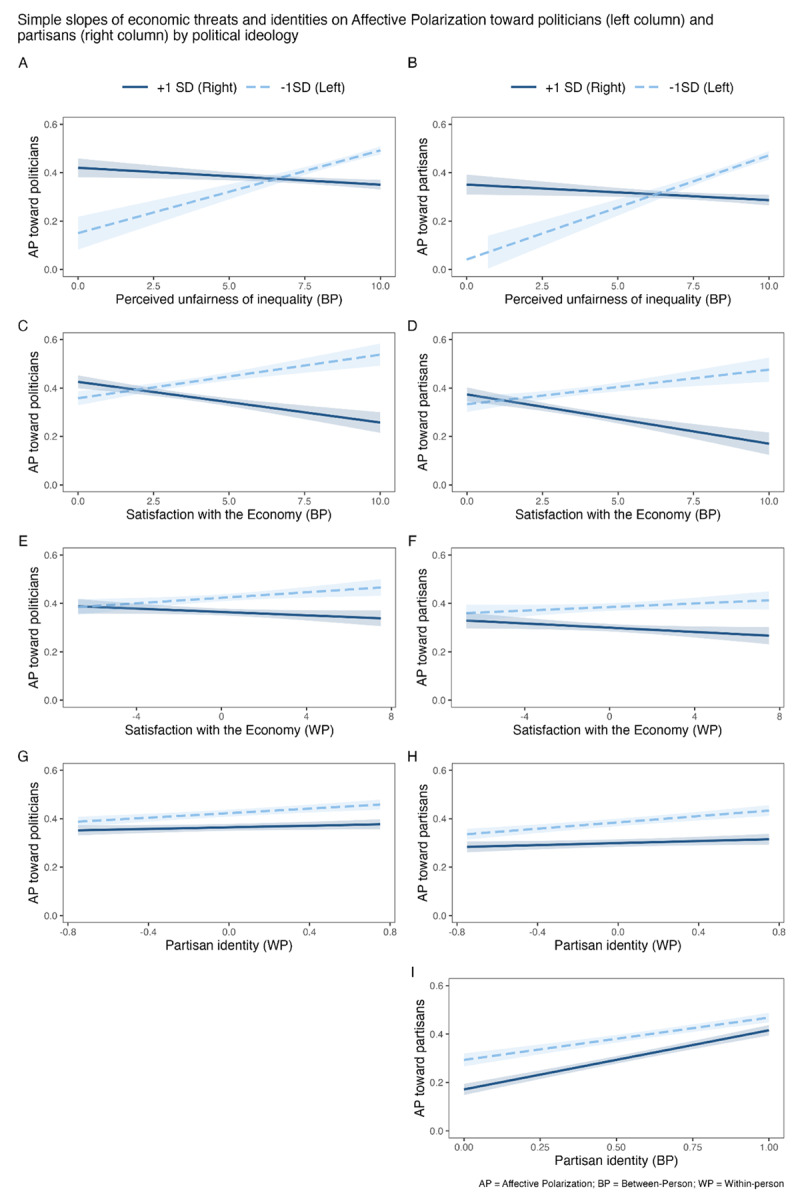
Simple slopes of economic threats and identities on affective polarization toward politicians and partisans by political ideology.

An overall interaction test, including all the interactions in a single model, suggests that political ideology moderated the relationship between affective polarization (toward politicians and partisans) and collective economic threats (i.e., perceived unfairness of the wealth distribution and satisfaction with the economy) (see Table S6 in the Supplementary Material). That is, among left-wing participants (1 SD below the mean), between-person perceived unfairness of wealth distribution (*b_Politicians_=* 0.041, *p <* .001; *b_Partisans_* = .043, *p <*.001) and satisfaction with the Spanish economy (*b_Politicians_ =* 0.023, *p <* .001; *b_Politicians_* = .014, *SE* = .004, *p <*.001) positively predicted affective polarization. On the contrary, among right-wing participants (1 SD above the mean), between-level perceived unfairness of wealth distribution (*b_Politicians_ = –*0.040, *p <* .001; *b_Partisans_* = –.006, *p* = .022) and satisfaction with the economy (*b_Politicians_ = –*0.045, *p <* .001; *b_Partisans_* = –.020, *p <*.001) negatively predicted affective polarization (see Figure 2, Panels A, B, C, and D).

Furthermore, at the within-person level, satisfaction with the economy positively predicted affective polarization among left-wing participants (toward politicians, *b_Politicians_* = .006, *p* = .008; and the evidence toward partisans was suggestive, *b_Partisans_* = .005, *p* = .066). On the contrary, among right-wing participants, an increase in satisfaction with the economy over time (within-person changes) negatively predicted affective polarization (particularly toward partisans, *b_Partisans_* = –.004, *p* = .044; and suggestively toward politicians, *b_Politicians_* = –.003, *p* = .082) (see Figure 2, Panels E and F).

Another consistent finding was that increasing partisan identity (within-person) over time positively predicted affective polarization among left-wing people (*b_politicians_* = 0.047, *p* < .001; *b_Partisans_* = 0.065, *p* < .001), but not among right-wing people (*b_politicians_* = 0.017, *p* = .101; *b_Partisans_* = 0.021, *p* = .057) (see Figure 2, Panels G and H).

Finally, the more people endorsed their political identity, the more affective polarization they showed toward partisans along all ideological lines, being stronger for right-wing participants (*b* = 0.244, *p* < .001) than for left-wing participants (*b* = 0.175, *p* < .001) (see Figure 2, panel I). Moderations inconsistent with affective polarization toward partisans and politicians are shown in the supplementary material (see section 7).

## Discussion

Economic threats, identities, and ideologies can fuel affective polarization. This general claim, however, requires further elaboration about what kinds of threats and identities influence affective polarization and how ideology might shape their effects. We aimed to test the role of subjective personal and collective economic threats and different identities in affective polarization in Spain. We also examined the consistency between different target groups (politicians and partisans), separated between-person differences from within-person changes over time, and explored whether such a relationship is shaped by political ideology.

### Personal economic threats on affective polarization

Regarding our first hypotheses, we found no significant evidence that between-person differences in personal economic threats were related to affective polarization. This finding was unexpected and contradicts the belief that individuals under high (vs. low) personal economic threat are likely to show greater affective polarization. One explanation is that the influence of personal economic concerns may be at the within-person level (but not at the between-person level), as we found that perceiving one personal economic situation as worsening over time was linked to greater affective polarization toward partisans but not politicians. This within-person economic decay may allow individuals to compare with their past selves, increasing their motivation to seek support from political or non-political social groups to deal with discomfort (e.g., political parties or social movements). Therefore, affective polarization may be a recursive process through which people who feel threatened engage with social groups, and such group identification exacerbates threat perceptions and animosities. Further research could examine whether economic identification with political groups explains the effect of economic threats on affective polarization.

Another explanation is that personal economic threats may have different effects if they focus on objective or subjective domains. Our measure of personal economic hardship relates more to objective indicators, as it asks about household income satisfaction, while personal economic concerns cover subjective preoccupations with different issues. Exploratory analyses testing each personal economic threat separately (see supplemental materials, Section 8) suggest that the less personal economic hardship (between-person), the more affective polarization toward politicians but not toward partisans. This result aligns with the effect of household income since the better-off showed greater polarization toward politicians than the worst-off. Our findings are congruent with the ‘fear of falling’ idea that wealthier classes show special angst about losing their status under uncertain economic scenarios (Jetten et al., 2017), facilitating the rise of affective polarization. Indeed, economic inequality increases people’s concerns about losing their privileges, fostering competition and zero-sum beliefs (Davidai & Tepper, 2023) that fuel polarization. Thus, subjective personal threats may make wealthy people feel threatened, despite not having objective economic constraints. Further research could focus on distinct sources of subjective personal economic threats for different groups along socioeconomic and ideological lines.

### Collective economic threats on affective polarization

Regarding our second hypothesis, our results supported the idea that collective economic threats are positively associated with affective polarization toward politicians and partisans. People were more polarized toward politicians and partisans when unsatisfied with the Spanish economy. Similarly, perceiving unfairness in wealth distribution was positively linked to greater affective polarization toward partisans (but not toward politicians). Interestingly, perceived unfairness in wealth distribution was the only collective economic threat that displayed a consistent longitudinal within-person positive effect on affective polarization.

The association between collective economic threats and affective polarization can be explained by the motivated social cognition model of economic threats (Fritsche & Jugert, 2017). This model proposes that collective economic threats lead people to use group-based or collective responses (instead of individual) to protect their self-esteem and restore control over the threatening situation (Klackl et al., 2022). One mechanism to recover control and cope with threats is identifying with social groups that fulfill individuals’ sense of control and belongingness (Fritsche et al., 2011). Thus, threats may motivate identification with some social groups that validate people’s feelings, exacerbating animosities and intergroup biases toward other groups (Comellas & Torcal, 2023; Iyengar et al., 2019). Since blaming other social groups and intergroup conflict are common responses after economic crises (Funke et al., 2016), we argue that perceived collective economic threats can leverage some social group identities that foster affective polarization.

Importantly, results from hypotheses 1 and 2 suggest that affective polarization was consistently associated with collective economic threats but not with personal economic threats. This difference can reflect different coping strategies to deal with the discomfort of threats: collective threats demand collective-based strategies, such as group identification or collective action, and individual threats elicit individual responses, such as ideological justifications or cognitive biases (Fritsche & Jugert, 2017).

We also speculate that a social identity analysis of economic inequality can also explain why collective economic threats fuel affective polarization. Negative evaluations of economic performance and aversion to inequality reinforce class-based identities, promoting intergroup biases (Tanjitpiyanond et al., 2022). As such, perceptions and evaluations of inequality can be seen as collective economic threats, hardening people’s negative attitudes toward other groups (Jetten et al., 2021) and exacerbating status anxiety and social distrust that end up undermining social cohesion (Goya-Tocchetto & Payne, 2021). Our results may suggest that perceiving unfairness in wealth distribution fosters a sense of competition (Sommet & Elliot, 2023), which may increase animosities toward partisans—but not politicians. Still, these explanations are speculative and should be tested formally in a design that allows extending the model of affective polarization linked to political groups to other non-political groups (e.g., socioeconomic groups).

### Political and Territorial Identities on Affective Polarization

Regarding the role of identities, we confirmed that political identity increased affective polarization. Indeed, the association between political identity and affective polarization is held at the between- and within-person level. These findings confirmed that political identity exerts a unique and important influence on fueling affective polarization (Comellas & Torcal, 2023; Dias & Lelkes, 2021; Iyengar et al., 2012, 2019). Of note, the effect size of political identity was more than three times bigger than that of national identity^5^ and about six to eight times bigger than the effect size of collective economic threats.^6^ Although this result reinforces the prominent role of political identity in affective polarization, it also makes clear that other sources of variance are unexplained by political identities. Our findings suggest that economic threats and territorial identities can also play an important role in explaining affective polarization.

We also confirmed our hypothesis that national identity was positively associated with affective polarization, the effect size being almost twice as large when it was toward politicians than toward partisans. The effect of national identity on affective polarization can indicate the competition between partisans over how to rule the nation regarding values and policies. Therefore, national identity leads people to engage in group-level mindsets that exacerbate collective economic and cultural threats (Klackl et al., 2022), which can undermine democracy (Comellas & Torcal, 2023). Our findings contrast previous research arguing that supraordinate national identities would reduce affective polarization (Levendusky et al., 2018). However, this national identity effect may be context-dependent, as it has not been successfully replicated (Brandt & Turner-Zwinkels, 2020). Thus, we argue that national identity will likely induce affective polarization in Spain because it has often been managed within right-wing political agendas that oppose egalitarian policies.

Regarding the role of regional identity, we found that it did not predict affective polarization. However, exploratory analyses suggested that political ideology can moderate this relationship. Regional identification led to greater polarization among right-wing participants and weaker polarization among left-wing participants. This exploratory result can reflect the nature of the political conflict about territorial identities in Spain (Orriols & León, 2020). Given that right-wing political ideology is related to greater levels of nationalism (Wojcieszak & Garrett, 2018), it is likely that regional identity becomes more relevant for right-wing individuals (vs. left-wing) because their identity is under threat as a result of independentist movements (e.g., Catalonia), weak governance (e.g., impeached government), and exacerbated territorial conflicts based on regional identities (Rodríguez et al., 2022). In Spain, there are right-wing and left-wing regionalist parties, indicating that regional and partisan identities account for unique sources of variance in affective polarization. Indeed, affective polarization in Spain has been partly aroused by managing national and regional identities along the political spectrum (Hernández et al., 2021).

On the other hand, European identity did not predict affective polarization, indicating that polarization can be driven more by between- and within-country characteristics (Bettarelli et al., 2022) than by supraordinate identities such as European belongingness. However, exploratory research in the supplementary material (section 8) suggests that European identity could positively predict affective polarization toward politicians once we excluded political partisanship from the model. This indicates that political identity may explain the potential effect of European identity. Still, this exploratory finding could also suggest that people who identify with Europe show more affective polarization because they may feel threatened by right-wing Eurosceptic parties (Down & Han, 2021). Further research should delve deeper into the interplay between different identities and beliefs to disentangle how they fuel polarization.

Two additional descriptive findings are noteworthy. One is that affective polarization toward politicians was higher than toward partisans. This result is consistent with previous research showing that people show greater negative feelings toward politicians than toward partisans (Druckman & Levendusky, 2019) and actors in non-political settings (Kingzette, 2021). Although partisans, understood as people voting for different parties, reflect political groups, they are also ordinary people exercising their voting rights and can support the party they stem from the most. For example, people from the same household can vote for different parties and still have frequent and positive relationships among them. This close contact with partisans (vs. politicians) may buffer people’s animosities and reduce polarization (Voelkel et al., 2022). Differentiating between targets in affective polarization can highlight the distinct consequences of polarization: polarization toward politicians threatens democracy, while polarization toward partisans threatens social cohesion.

The other descriptive finding is that affective polarization slightly increased over the 6-month period of the study. This finding is consistent with Spain’s agitated political climate during data collection due to electoral campaigns. By writing this article, Spain faced another critical political time to form a new government after the 2023 general elections. Further research should continue analyzing how affective polarization evolves over time and how people change their views before these critical times.

### Limitations and further research

One of the limitations of our study is that different measures of affective polarization can capture different dimensions of the same construct (Lelkes, 2016). We used a measure that accounts for the dispersion of feelings about different groups in multiparty systems (Wagner, 2021). We double-checked the robustness of our results by using several variations of our affective polarization measure (with more political actors, using weighted and unweighted versions of the scale), which showed no substantial differences. Alternative measures could also be examined to shed light on alternative research questions, such as splitting between in-group and out-group affects for comparing whether polarization is driven by liking or disliking different groups or examining affective polarization toward left- or right-wing social groups (Rodríguez et al., 2022). Further research should try to disentangle what specific dimensions of affective polarization can be captured by alternative measures.

Another limitation is that perceived collective economic threats can be conflated with ideological attitudes: satisfaction with the economy and perceived unfairness in wealth distribution may be related to system-justifying beliefs and attitudes toward redistribution, respectively. We could not influence the item selection as we relied on secondary data. Still, we reason that these items capture a sense of threat, as they indicate something is going wrong with participants’ personal and societal economic situation. For instance, Kahn et al. (2022) found that people identified justice-related issues as a specific global threat affecting society. Stephan et al. (2015) argue that group-level threats are concerns about losing material resources or cultural values, which are implicit ideas in people’s perceptions of unfairness. That is, unfair perceptions of inequality indicate something is wrong, signaling potential collective economic harm (Jost et al., 2022; Reiss et al., 2021). Furthermore, single-item indicators are more prone to measurement error. Therefore, further research should clearly distinguish between subjective perceptions of inequality and ideological beliefs and use composite measures or latent variable modeling techniques.

A final caveat of our study is that the results may vary according to people’s political ideologies. On the one hand, we found that the more perceived unfairness of wealth distribution, the more affective polarization among left-wing participants. On the other hand, the greater the satisfaction with the economy, the less affective polarization there is among right-wing participants. Although these results are exploratory, they may suggest that collective economic threats increase affective polarization for both left- and right-wing participants, but for different motives: left-wing participants were more concerned about unfairness evaluations that threaten their core values, while right-wing participants were more concerned about the stability of the status quo (Jost et al., 2022). Unexpectedly, left-wing participants showed a positive association between satisfaction with the economy and affective polarization. We speculate this counterintuitive finding is because left-wing people may show strong emotional reactions when a well-functioning economy does little to distribute wealth in a more egalitarian way. Another explanation is that the salience of elections during the study’s data collection period made left-wing participants who were satisfied with the economy more responsive to emerging right-wing political campaigns demanding economic reforms. These exploratory findings are aligned with previous research showing that reactions to threats depend on the type of threat (Brandt et al., 2021), ideological differences (Fiagbenu & Kessler, 2022), and the salience of elections (Hernández et al., 2021).

In brief, our findings confirmed extensive literature assuring that political identity is one of the most powerful predictors of affective polarization. However, we also signaled the relevance of subjective collective economic threats and national identity in exacerbating animosities toward different political actors. Affective polarization seems to result from several forces at different levels (e.g., economic, political, social, intergroup, etc.), which converge in how people make sense of the world, other groups, and themselves (Jost et al., 2022). Thus, the interplay between threats, identities, and ideologies provides an additional layer that could broaden our understanding of affective polarization and its potential consequences for our societies.

## Additional File

The additional file for this article can be found as follows:

10.5334/irsp.838.s1 Supplementary Material. Supplemental materials include descriptive results, robustness checks, and exploratory analyses. Data and materials used in this manuscript are publicly available at https://osf.io/sw8je/.

## Footnotes

According to census data from Spain on October 29th, 50.95% were female and 41.05% were male.

The formula to rescale the variable Scaled = (value – min. value)/(max. value – min. value).

PP = Partido popular (traditional right-wing), PSOE = Partido Socialista Obrero Español (traditional left-wing), Cs = Ciudadanos (renewed right-wing), and UP = Unidas podemos (renewed left-wing).

The weighting scores were: PP = 0.52), PSOE = 0.27, Cs = 0.06, and UP = 0.15. These weights were calculated based on data available at: for deputies, https://www.congreso.es/grupos/composicion-en-la-legislatura; for senators, https://www.senado.es/web/composicionorganizacion/gruposparlamentarios/gruposparlamentariosdesde1977/index.html.

Based on the ratio between *b_political-identity_/b_national-identity_*, affective polarization toward politicians is 0.364/0.111, and toward partisans, it is 0.363/0.053.

Based on the ratio between *b_political-identity_/b_collective-threat_*, affective polarization toward politicians is 0.364/–0.042, and toward partisans, it is 0.363/–0.069
