# Supplementary material for: Economic Threats, Political and National Identification Predict Affective Polarization: Longitudinal Evidence From Spain
Source: Int Rev Soc Psychol. 2024 Mar 21;37:5. doi: 10.5334/irsp.838 (PMC12372765; doi:10.5334/irsp.838)
Supplement: Supplementary Material. — Supplemental materials include descriptive results, robustness checks, and exploratory analyses. Data and materials used in this manuscript are publicly available at https://osf.io/sw8je/. [file irsp-37-838-s1.pdf]

**Supplemental material of:**

# Economic threats, political and national identification predict affective polarization: Longitudinal evidence from Spain

[AUTHORS ANONYMIZED FOR PEER REVIEW]

## Table of content

1. Descriptive statistics of the variables included in the study per wave........................................................

2. Attrition analyses ........................................................................................................................

3. Robustness checks: Main analysis using another proxy of political identity ..........................................

4. Robustness checks: Main analyses using alternative variations of affective polarization toward political elites and partisans........................................................................................................................

5. Model Diagnostics ........................................................................................................................

6. Exploratory analyses: Interactions tested Diagnostics........................................................................

7. Exploratory analyses: Simple slopes analyses for interactions non-consistent across measures of affective polarization ........................................................................................................................

8. Robustness checks: Testing personal economic threats separately ........................................................

9. Robustness checks: Testing identities separately ..............................................................................

# 1. Descriptive statistics of the variables included in the study per wave

Table S1.1 Descriptive statistics of the variables included in the study per wave

| | Wave 1 | | | Wave 2 | | | Wave 3 | | | Wave 4 | | |
|---|---|---|---|---|---|---|---|---|---|---|---|---|
| | **n** | **mean** | **sd** | **n** | **mean** | **sd** | **n** | **mean** | **sd** | **n** | **mean** | **sd** |
| 1. Affective polarization toward politicians | 2478 | 0.41 | 0.26 | 1879 | 0.40 | 0.27 | 1638 | 0.43 | 0.27 | 2043 | 0.41 | 0.27 |
| 1a. Feelings towards Pablo Casado [PP] | 2497 | 26.92 | 27.84 | 1889 | 27.80 | 27.79 | 1653 | 29.83 | 28.46 | 2051 | 30.00 | 27.22 |
| 1b. Feelings towards Pedro Sánchez [PSOE] | 2494 | 40.74 | 28.41 | 1886 | 40.59 | 29.14 | 1653 | 45.11 | 29.50 | 2057 | 47.58 | 29.16 |
| 1c. Feelings towards Albert Rivera [Cs] | 2494 | 35.61 | 30.52 | 1885 | 35.15 | 29.27 | 1657 | 38.97 | 29.97 | 2054 | 38.04 | 29.40 |
| 1d. Feelings towards Pablo Iglesias [UP] | 2494 | 35.15 | 30.51 | 1889 | 34.03 | 28.95 | 1651 | 42.57 | 31.34 | 2055 | 41.89 | 31.09 |
| 2. Affective polarization toward partisans | 2475 | 0.36 | 0.28 | 1879 | 0.36 | 0.28 | 1651 | 0.37 | 0.29 | 2043 | 0.37 | 0.29 |
| 2a. Feelings towards PP voters | 2490 | 36.25 | 29.51 | 1888 | 35.49 | 28.68 | 1656 | 38.37 | 28.85 | 2054 | 38.59 | 28.68 |
| 2b. Feelings towards PSOE voters | 2493 | 51.43 | 24.86 | 1888 | 51.70 | 24.52 | 1655 | 54.48 | 25.79 | 2055 | 55.46 | 25.85 |
| 2c. Feelings towards Cs voters | 2496 | 41.81 | 29.99 | 1883 | 41.00 | 28.25 | 1655 | 44.22 | 28.54 | 2057 | 43.52 | 28.11 |
| 2d. Feelings towards UP voters | 2495 | 46.32 | 30.54 | 1890 | 45.66 | 29.27 | 1657 | 51.98 | 30.75 | 2054 | 50.67 | 30.63 |
| 3. Personal economic concerns | 2501 | 1.37 | 0.79 | 1890 | 1.35 | 0.80 | 1890 | 1.35 | 0.83 | 2059 | 1.35 | 0.79 |
| 3a. Concern about bills | 2501 | 1.42 | 0.96 | 1890 | 1.42 | 0.97 | 1659 | 1.45 | 0.97 | 2059 | 1.41 | 0.95 |
| 3b. Concern about reducing lifestyle | 2501 | 1.43 | 0.88 | 1890 | 1.37 | 0.90 | 1659 | 1.42 | 0.92 | 2059 | 1.40 | 0.89 |
| 3c. Concern about getting a job | 2501 | 1.50 | 1.09 | 1890 | 1.48 | 1.10 | 1659 | 1.50 | 1.10 | 2059 | 1.49 | 1.09 |
| 3d. Concern about loans and mortgages | 2501 | 1.12 | 1.06 | 1890 | 1.13 | 1.05 | 1659 | 1.16 | 1.08 | 2059 | 1.11 | 1.05 |
| 4. Personal economic hardship | 2501 | 1.00 | 0.84 | 1890 | 1.00 | 0.83 | 1659 | 1.04 | 0.83 | 2059 | 1.02 | 0.81 |
| 5. Satisfaction with Spanish economy | 2498 | 3.25 | 2.25 | 1890 | 3.62 | 2.25 | 1659 | 3.77 | 2.23 | 2059 | 3.95 | 2.22 |
| 6. Perceived unfairness of wealth distribution | 2498 | 8.16 | 2.50 | 1889 | 8.02 | 2.54 | 1659 | 7.88 | 2.59 | 2058 | 7.83 | 2.63 |
| 7. Regional identity | 2497 | 7.28 | 2.65 | 1884 | 7.41 | 2.61 | - | - | - | - | - | - |
| 8. National identity | 2497 | 6.75 | 3.08 | 1884 | 6.84 | 3.12 | - | - | - | - | - | - |
| 9. European identity | 2491 | 6.46 | 2.60 | 1885 | 6.46 | 2.65 | - | - | - | - | - | - |
| 10. Partisan identity | 2498 | 0.45 | 0.50 | 1888 | 0.50 | 0.50 | 1657 | 0.55 | 0.50 | 2058 | 0.57 | 0.49 |
| 11. Political ideology | 2499 | 4.02 | 2.35 | 1828 | 3.98 | 2.41 | 1659 | 3.92 | 2.53 | - | - | - |
| 12. Media consumption | 2403 | 0.86 | 1.48 | 1813 | 0.81 | 1.53 | 1644 | 0.90 | 1.66 | 2033 | 1.21 | 1.88 |
| 13. Political interest | 2499 | 2.74 | 0.84 | 1888 | 2.75 | 0.81 | 1659 | 2.73 | 0.80 | 2059 | 2.70 | 0.82 |
| 14. Residence (urban) | 2501 | 0.17 | 0.38 | 2501 | 0.17 | 0.38 | 2501 | 0.17 | 0.38 | 2501 | 0.17 | 0.38 |
| 15. Sex (Female) | 2501 | 0.49 | 0.50 | 2501 | 0.49 | 0.50 | 2501 | 0.49 | 0.50 | 2501 | 0.49 | 0.50 |
| 16. Education | 2501 | 5.06 | 1.47 | 2501 | 5.06 | 1.47 | 2501 | 5.06 | 1.47 | 2501 | 5.06 | 1.47 |
| 17. Income | 2501 | 5.19 | 2.84 | - | - | - | - | - | - | - | - | - |

Note: Blank spaces (-) mean that the variable was not surveyed in that wave; PP = Partido Popular (traditional right); PSOE = Partido Socialista Obrero Español (traditional left); Cs = Ciudadanos (new right); UP = Unidas Podemos (new left).

Table S1.2. Descriptive statistics and Pearson correlations between variables included in the Study

| | 1 | 2 | 3 | 4 | 5 | 6 | 7 | 8 | 9 | 10 | 11 | 12 | 13 | 14 | 15 | 16 | 17 |
|---|---|---|---|---|---|---|---|---|---|---|---|---|---|---|---|---|---|
| 1. Affective polarization toward political elites | | | | | | | | | | | | | | | | | |
| 2. Affective polarization toward partisans | .788*** | | | | | | | | | | | | | | | | |
| 3. Personal economic concerns | -.070*** | -.022 | | | | | | | | | | | | | | | |
| 4. Personal economic hardship | -.053** | -.010 | .602*** | | | | | | | | | | | | | | |
| 5. Satisfaction with Spanish economy | .064** | -.006 | -.276*** | -.311*** | | | | | | | | | | | | | |
| 6. Perceived unfairness of wealth distribution | .084*** | .133*** | .124*** | .196*** | -.205*** | | | | | | | | | | | | |
| 7. Regional identity | .122*** | .103*** | -.052** | -.037 | .061** | .097*** | | | | | | | | | | | |
| 8. National identity | .070*** | -.037 | -.013 | -.038 | .193*** | -.181*** | .286*** | | | | | | | | | | |
| 9. European identity | .099*** | .020 | -.132*** | -.154*** | .273*** | -.130*** | .376*** | .491*** | | | | | | | | | |
| 10. Partisan identity | .504*** | .487*** | -.012 | .002 | .101*** | .067*** | .145*** | .000 | .098*** | | | | | | | | |
| 11. Political ideology | -.144*** | -.212*** | -.039 | -.076*** | .084*** | -.415*** | -.021 | .371*** | .166*** | -.125*** | | | | | | | |
| 12. Media consumption | .144*** | .180*** | .055** | .017 | .032 | -.028 | -.015 | -.115*** | -.039 | .226*** | -.038 | | | | | | |
| 13. Political interest | .384*** | .338*** | -.075*** | -.063** | .189*** | -.005 | .097*** | -.017 | .133*** | .451*** | -.071*** | .382*** | | | | | |
| 14. Residence (Rural) | .006 | .015 | .051* | .046* | -.047* | .045* | -.024 | -.058** | -.060** | -.015 | -.043* | -.022 | -.032 | | | | |
| 15. Sex (Female) | -.101*** | -.054** | .173*** | .059** | -.179*** | .047* | .029 | -.026 | -.005 | -.043* | -.058** | -.099*** | -.245*** | .037 | | | |
| 16. Education | -.002 | -.024 | -.170*** | -.265*** | .119*** | -.101*** | -.056** | -.072*** | .122*** | .012 | .024 | .025 | .154*** | -.081*** | .048* | | |
| 17. Income | .125*** | .067*** | -.351*** | -.437*** | .177*** | -.143*** | .063** | .024 | .124*** | .060** | .057** | .025 | .130*** | -.028 | -.104*** | .245*** | |
| Mean | 0.41 | 0.36 | 1.36 | 2.01 | 3.60 | 8.00 | 7.32 | 6.78 | 6.47 | 0.50 | 4.02 | 0.92 | 2.73 | 0.17 | 0.49 | 5.06 | 5.19 |
| SD | 0.24 | 0.25 | 0.73 | 0.75 | 1.94 | 2.11 | 2.46 | 2.95 | 2.44 | 0.43 | 2.35 | 1.35 | 0.76 | 0.38 | 0.50 | 1.47 | 2.84 |

Note: ***p<.001, **p<.01, *p<.05

***Figure S1.*** *Affective polarization toward politicians (panel A) and partisans (panel B) across four waves. Note: Within-person patterns are shown in gray, aggregated values per wave in red dots, and the regression line at the between-person level is depicted in blue.*

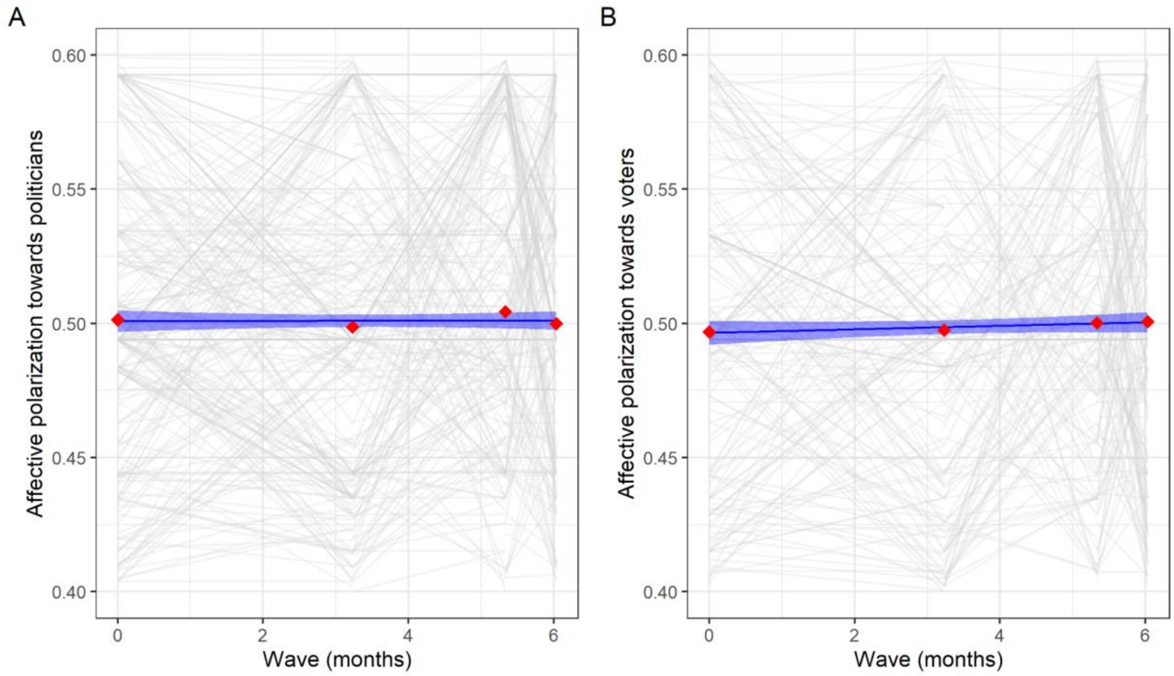

## 2. Attrition analyses

The attrition rate between waves 1 and 2 was 24.43%; between waves 2 and 3 was 12.22%, and between waves 3 and 4 was 10.55%. Attrition analyses revealed that sex, area of residence (urban vs. rural), education level, household income, and political ideology were uncorrelated with remaining in the panel (see Supplementary Material Table S2). The focal variables of this study were uncorrelated with remaining in the panel, except for perceived unfairness of wealth redistribution (b = 2.56, $p$ = .035, OR = .94), and European identity (b = 2.56, $p$ = .034, OR = .94). Similarly, age was positively associated with remaining in the panel in waves 3 to 4 (b = .01, $p$ = .036, OR = 1.01). Because of the relatively small effect size of these associations and high p-values, we assume that the sample was not severely biased.

Table S2. Logistic regression for participating in the survey over the study waves (attrition analysis)

| | **Completed wave 2 (nested in wave 1)** | | **Completed wave 3 (nested in waves 2 and 1)** | | **Completed wave 4 (nested only in wave 1)** | | **Completed all waves (w3, nested in w2 and w1)** | |
|---|---|---|---|---|---|---|---|---|
| *Predictors* | *OR* | *p* | *OR* | *p* | *OR* | *p* | *OR* | *p* |
| (Intercept) | 9.58 (6.52) | **.001** | 3.52 (2.15) | **.039** | 1.75 (1.35) | .465 | 2.73 (1.57) | .082 |
| Gender (Female) | 1.02 (0.13) | .890 | 0.99 (0.12) | .939 | 0.86 (0.13) | .316 | 0.94 (0.10) | .586 |
| Age | 1.00 (0.01) | .764 | 1.01 (0.00) | **.004** | 1.00 (0.01) | .676 | 1.01 (0.00) | **.036** |
| Rural (vs. Urban) | 1.04 (0.18) | .803 | 1.13 (0.17) | .426 | 1.33 (0.27) | .165 | 1.25 (0.18) | .119 |
| Education | 0.91 (0.04) | .054 | 0.91 (0.04) | **.030** | 1.09 (0.06) | .117 | 0.95 (0.04) | .244 |
| Income | 1.02 (0.03) | .549 | 0.97 (0.02) | .244 | 0.99 (0.03) | .842 | 0.96 (0.02) | .080 |
| Political ideology | 0.98 (0.03) | .429 | 0.98 (0.03) | .441 | 1.06 (0.04) | .105 | 1.00 (0.02) | .987 |
| Personal economic hardship | 1.07 (0.12) | .554 | 1.16 (0.12) | .163 | 1.06 (0.14) | .659 | 1.11 (0.11) | .287 |
| Personal economic concerns | 1.00 (0.11) | .970 | 1.00 (0.10) | .993 | 1.01 (0.14) | .924 | 0.98 (0.10) | .847 |
| Satisfaction with the Spanish Economy | 1.01 (0.04) | .696 | 1.04 (0.03) | .215 | 1.05 (0.04) | .272 | 1.03 (0.03) | .327 |
| Perceived unfairness of the wealth distribution | 0.95 (0.03) | .130 | 0.93 (0.03) | **.026** | 1.01 (0.04) | .724 | 0.94 (0.03) | **.035** |
| Identity: partisanship | 1.00 (0.12) | .999 | 1.05 (0.12) | .675 | 1.12 (0.16) | .406 | 1.05 (0.11) | .655 |
| Identity: national | 1.00 (0.03) | .881 | 1.01 (0.02) | .609 | 1.00 (0.03) | .925 | 1.01 (0.02) | .781 |
| Identity: regional | 1.02 (0.03) | .497 | 1.01 (0.03) | .702 | 1.05 (0.03) | .115 | 1.01 (0.02) | .569 |
| Identity: European | 0.94 (0.03) | **.049** | 0.94 (0.03) | **.034** | 0.93 (0.03) | .054 | 0.94 (0.03) | **.015** |
| Observations | 1605 | | 1605 | | 1605 | | 1605 | |
| $R^2$ Tjur | 0.009 | | 0.025 | | 0.008 | | 0.020 | |

*Note*. OR = Odds Ratio

### 3. Robustness checks: Main analysis using another proxy of political identity

For robustness checks, we replaced the dichotomous variable of political identity reported in the main manuscript (i.e., "Do you consider yourself close to any political party?") with an alternative proxy item that quantifies the level of closeness that people feel toward their political party (i.e. "And how close do you feel to this party?"). The response scale for this proxy indicator used a 4-point Likert scale: 3 "Very close", 2 "Somewhat close", 1 "Not very close", and 0 "Not at all close".

As shown in Table S3 (see bolded and underlined coefficients), we obtained similar results to those reported in the main manuscript. That is, political identity (as level of closeness to a political party) was positively associated (both at the within- and between-person level) with affective polarization toward politicians and partisans.

Table S3. Multilevel regression analysis using an alternative proxy of political identity

| | **Affective polarization toward politicians** | **Affective polarization toward partisans** |
|---|---|---|
| Predictors | *β (SE)* | *β (SE)* |
| (Intercept) | -0.036<br>(0.023) | -0.027<br>(0.023) |
| Time (months) | 0.002<br>(0.011) | -0.004<br>(0.011) |
| *Covariates* | | |
| Age | 0.084 $^{**}$<br>(0.026) | 0.002<br>(0.026) |
| Sex (Female) | -0.001<br>(0.025) | 0.013<br>(0.025) |
| Education, 8 levels | -0.036<br>(0.025) | -0.055 $^{*}$<br>(0.026) |
| Income | 0.020<br>(0.026) | -0.006<br>(0.026) |
| Political ideology (left-right) | -0.121 $^{***}$<br>(0.030) | -0.133 $^{***}$<br>(0.030) |
| Rural (vs. urban) | 0.032<br>(0.023) | -0.003<br>(0.023) |
| Media consumption (between-person) | -0.024<br>(0.025) | 0.040<br>(0.025) |
| Media consumption (within-person) | 0.011<br>(0.011) | -0.005<br>(0.011) |
| Political interest (between-person) | 0.166 $^{***}$<br>(0.026) | 0.106 $^{***}$<br>(0.026) |
| Political interest (within-person) | 0.023 $^{*}$<br>(0.011) | 0.022 $^{*}$<br>(0.011) |

*Continued…*

Table S3. *Continued*

| Predictors | Affective polarization toward politicians<br>*β (SE)* | Affective polarization toward partisans<br>*β (SE)* |
|---|---|---|
| *Within-person level* | | |
| Personal economic hardship | 0.012 | 0.021 |
| | (0.011) | (0.011) |
| Personal economic concerns | -0.002 | 0.002 |
| | (0.011) | (0.011) |
| Satisfaction with economy | 0.005 | -0.002 |
| | (0.011) | (0.011) |
| Perceived unfairness of wealth redistribution | 0.021 * | 0.011 |
| | (0.011) | (0.011) |
| **Political identity** | **0.051 ***** | **0.043 ***** |
| | **(0.010)** | **(0.010)** |
| *Between-person level* | | |
| Personal economic hardship | -0.059 | -0.047 |
| | (0.032) | (0.032) |
| Personal economic concerns | 0.004 | -0.014 |
| | (0.031) | (0.031) |
| Satisfaction with economy | -0.048 | -0.076 ** |
| | (0.026) | (0.026) |
| Perceived unfairness of wealth redistribution | 0.015 | 0.047 |
| | (0.028) | (0.028) |
| **Political identity** | **0.204 ***** | **0.206 ***** |
| | **(0.023)** | **(0.023)** |
| Regional identity | -0.002 | 0.013 |
| | (0.024) | (0.024) |
| National identity | 0.161 *** | 0.121 *** |
| | (0.029) | (0.029) |
| European identity | -0.026 | -0.051 |
| | (0.027) | (0.028) |
| Random Effects | | |
| $\sigma^2$ | 0.022 | 0.028 |
| $\tau_{00}$ | 0.032 | 0.039 |
| N (participants) | 1365 | 1361 |
| N (Regions) | 17 | 17 |
| Observations | 3627 | 3642 |
| Marginal $R^2$ / Conditional $R^2$ | 0.251 | 0.227 |

* p<0.05 ** p<0.01 *** p<0.001

## 4. Robustness checks: Main analyses using alternative variations of affective polarization toward political elites and partisans

Considering that affective polarization measures can be conceptualized in different ways, we performed the main analyses of the manuscript using several variations of the outcome variable. In Table S4, M1a are the results reported in the main manuscript, in which affective polarization was based on the leaders of the four main political parties, weighted by the size of the party in the Parliament. In M1b, we computed the unweighted version of the previous measure. As for M1c, we extended the measure of affective polarization by adding three political characters that were linked to separatist movements within Spain (i.e., Íñigo Urkullu from the Nationalist Basque Party, Carles Puigdemont and Oriol Junqueras, promoters of the referendum for the self-declared independence of Catalonia). As for Affective polarization toward partisans, M2a and M2b present the weighted and unweighted version of the people's feelings toward people that support the four main political parties when collecting the data (i.e., PSOE, PP, UP, and Cs). As shown in Table S4, the unweighted version and the extended measure of affective polarization toward political elites did not show substantive differences with the results reported in the main manuscript, with two exceptions. First, the association between personal economic hardship was not statistically significant in the weighted version, but negative and statistically significant in the unweighted version. In other words, when we discard the information about political party size for computing affective polarization, we found that experiencing lower economic hardship was related to greater affective polarization.

The second exception concerns the role of regional and national identities on affective polarization. We found that regional identity was positively related to affective polarization toward political elites when the measure included political characters linked to independentist agendas, while such an association was not significant in the weighted nor unweighted version of affective polarization. Furthermore, national identity was positively related to greater affective polarization (both weighted and unweighted) but was not associated with the extended measure of affective polarization. In other words, including political leaders who support the independence of some regions from Spain can exacerbate the role of regional identity instead of national identity. These findings might be due to the nature of the political conflict in Spain, which is mostly based on long-standing territorial issues and regional identities (Miller, 2020; Rojo, 2021). In all other respects, the results were virtually unchanged between the variations of the measures.

Table S4. Robustness checks: Multilevel regression analyses for different variations of affective polarization measures toward politicians and partisans

| | Affective polarization toward politicians | | | Affective polarization toward partisans | |
|---|---|---|---|---|---|
| | M1a: Reported (four main parties and weighted) | M1b: Alternative (for four main parties and unweighted) | M1c: Alternative (for seven parties and unweighted) | M2a: Reported (four parties and weighted) | M2b: Alternative (four parties and unweighted) |
| Predictors | *β (SE)* | *β (SE)* | *β (SE)* | *β (SE)* | *β (SE)* |
| (Intercept) | -0.000 | -0.001 | -0.018 | -0.001 | -0.002 |
| | (0.016) | (0.016) | (0.029) | (0.016) | (0.016) |
| Time (months) | 0.009 | 0.000 | 0.028 *** | 0.009 | -0.002 |
| | (0.007) | (0.007) | (0.007) | (0.007) | (0.008) |
| *Covariates* | | | | | |
| Age | 0.089 *** | 0.083 *** | 0.135 *** | 0.032 | 0.033 |
| | (0.018) | (0.018) | (0.018) | (0.019) | (0.018) |
| Sex (Female) | -0.020 | -0.022 | -0.013 | -0.004 | -0.010 |
| | (0.017) | (0.017) | (0.017) | (0.018) | (0.017) |
| Education, 8 levels | -0.018 | -0.016 | -0.017 | -0.032 | -0.025 |
| | (0.018) | (0.018) | (0.017) | (0.018) | (0.018) |
| Income | 0.046 * | 0.044 * | 0.055 ** | 0.020 | 0.018 |
| | (0.019) | (0.019) | (0.018) | (0.019) | (0.019) |
| Political ideology (left-right) | -0.094 *** | -0.048 * | -0.022 | -0.117 *** | -0.084 *** |
| | (0.020) | (0.020) | (0.019) | (0.020) | (0.020) |
| Rural (vs. urban) | 0.015 | 0.013 | 0.012 | 0.012 | 0.011 |
| | (0.016) | (0.016) | (0.016) | (0.016) | (0.016) |
| Media consumption (between-person) | -0.004 | -0.005 | -0.016 | 0.044 * | 0.046 ** |
| | (0.018) | (0.018) | (0.017) | (0.018) | (0.018) |
| Media consumption (within-person) | 0.021 ** | 0.020 ** | 0.027 *** | 0.009 | 0.008 |
| | (0.007) | (0.007) | (0.007) | (0.007) | (0.007) |
| Political interest (between-person) | 0.162 *** | 0.171 *** | 0.174 *** | 0.114 *** | 0.126 *** |
| | (0.020) | (0.020) | (0.020) | (0.021) | (0.020) |
| Political interest (within-person) | 0.021 ** | 0.020 ** | 0.027 *** | 0.027 *** | 0.027 *** |
| | (0.007) | (0.007) | (0.007) | (0.007) | (0.007) |
| *Economic Threats (within-person)* | | | | | |
| Personal economic hardship | 0.008 | 0.007 | 0.005 | 0.017 * | 0.019 ** |
| | (0.007) | (0.007) | (0.007) | (0.007) | (0.007) |
| Personal economic concerns | -0.001 | -0.001 | -0.001 | 0.004 | 0.003 |
| | (0.007) | (0.007) | (0.007) | (0.007) | (0.007) |
| Satisfaction with economy | 0.003 | -0.006 | -0.004 | -0.003 | -0.002 |
| | (0.007) | (0.007) | (0.007) | (0.007) | (0.007) |
| Perceived unfairness of wealth redistribution | 0.021 ** | 0.022 ** | 0.027 *** | 0.015 * | 0.018 * |
| | (0.007) | (0.007) | (0.007) | (0.007) | (0.007) |
| *Economic Threats (between-person)* | | | | | |
| Personal economic hardship | -0.037 | **-0.051 *** | **-0.064 **** | -0.025 | -0.029 |
| | (0.022) | **(0.022)** | **(0.022)** | (0.023) | (0.023) |
| Personal economic concerns | -0.005 | 0.007 | 0.011 | -0.016 | -0.017 |
| | (0.021) | (0.021) | (0.021) | (0.022) | (0.021) |
| Satisfaction with economy | **-0.042 *** | **-0.068 ***** | **-0.069 ***** | **-0.069 ***** | **-0.092 ***** |
| | **(0.018)** | **(0.018)** | **(0.018)** | **(0.019)** | **(0.018)** |
| Perceived unfairness of wealth redistribution | 0.019 | 0.022 | 0.034 | **0.038 *** | **0.040 *** |
| | (0.019) | (0.019) | (0.018) | **(0.019)** | **(0.019)** |
| *Identity (within-person)* | | | | | |
| Political identity | **0.033 ***** | **0.039 ***** | **0.042 ***** | **0.040 ***** | **0.043 ***** |
| | **(0.007)** | **(0.007)** | **(0.007)** | **(0.007)** | **(0.007)** |
| *Identity (between-person)* | | | | | |
| Political identity | **0.363 ***** | **0.361 ***** | **0.319 ***** | **0.363 ***** | **0.365 ***** |
| | **(0.018)** | **(0.019)** | **(0.018)** | **(0.019)** | **(0.019)** |
| Regional identity | **-0.003** | **0.013** | **0.128 ***** | 0.009 | 0.020 |
| | **(0.018)** | **(0.018)** | **(0.018)** | (0.018) | (0.018) |
| National identity | **0.117 ***** | **0.109 ***** | **-0.023** | **0.057 **** | **0.042 *** |
| | **(0.021)** | **(0.021)** | **(0.021)** | **(0.021)** | **(0.021)** |

| | | | | | |
|---|---|---|---|---|---|
| European identity | -0.025 | -0.025 | 0.018 | -0.035 | -0.039 |
| | (0.020) | (0.020) | (0.019) | (0.020) | (0.020) |
| Economic inequality | -0.011 | -0.010 | 0.008 | -0.024 | -0.023 |
| | (0.016) | (0.016) | (0.025) | (0.016) | (0.016) |
| Mean income per capita | 0.018 | 0.015 | 0.018 | 0.006 | 0.007 |
| | (0.017) | (0.017) | (0.030) | (0.017) | (0.017) |
| Random Effects | | | | | |
| $\sigma^2$ (Within-person variance) | 0.023 | 0.023 | 0.018 | 0.027 | 0.029 |
| $\tau_{00}$ (Between-person variance) | 0.029 | 0.029 | 0.020 | 0.035 | 0.034 |
| N (participants) | 2051 | 2051 | 2051 | 2047 | 2047 |
| N (regions) | 17 | 17 | 17 | 17 | 17 |
| Observations | 6933 | 6933 | 6899 | 6944 | 6944 |
| Marginal $R^2$ | 0.449 | 0.439 | 0.279 | 0.412 | 0.409 |

* p<0.05 ** p<0.01 *** p<0.001

## 5. Model Diagnostics

Table S5. Variance Inflation Factor for the final regression models reported in the manuscript

| Variable | Affective polarization toward politicians | | Affective polarization toward partisans | |
|---|---|---|---|---|
| | VIF | Tolerance | VIF | Tolerance |
| Time (months) | 1.11 | 0.90 | 1.11 | 0.90 |
| Age | 1.32 | 0.76 | 1.32 | 0.76 |
| Sex (Female) | 1.19 | 0.84 | 1.19 | 0.84 |
| Education, 8 levels | 1.27 | 0.79 | 1.27 | 0.79 |
| Income | 1.40 | 0.72 | 1.40 | 0.71 |
| Political ideology (left-right) | 1.54 | 0.65 | 1.54 | 0.65 |
| Rural (vs. urban) | 1.03 | 0.97 | 1.03 | 0.97 |
| Media consumption (between-person) | 1.24 | 0.81 | 1.24 | 0.81 |
| Media consumption (within-person) | 1.02 | 0.98 | 1.02 | 0.98 |
| Political interest (between-person) | 1.64 | 0.61 | 1.64 | 0.61 |
| Political interest (within-person) | 1.02 | 0.98 | 1.02 | 0.98 |
| Personal economic hardship (WP) | 1.03 | 0.97 | 1.03 | 0.97 |
| Personal economic concerns (WP) | 1.03 | 0.97 | 1.03 | 0.97 |
| Satisfaction with economy (WP) | 1.07 | 0.94 | 1.06 | 0.94 |
| Perceived unfairness of wealth redistribution (WP) | 1.01 | 0.99 | 1.01 | 0.99 |
| Identity (within-person) | 1.05 | 0.96 | 1.05 | 0.96 |
| Personal economic hardship (BP) | 1.99 | 0.50 | 2.00 | 0.50 |
| Personal economic concerns (BP) | 1.79 | 0.56 | 1.80 | 0.56 |
| Satisfaction with economy (BP) | 1.34 | 0.74 | 1.34 | 0.74 |
| Perceived unfairness of wealth redistribution (BP) | 1.41 | 0.71 | 1.41 | 0.71 |
| Identity (between-person) | 1.36 | 0.73 | 1.36 | 0.74 |
| Regional identity | 1.27 | 0.79 | 1.26 | 0.79 |
| National identity | 1.75 | 0.57 | 1.75 | 0.57 |
| European identity | 1.60 | 0.62 | 1.60 | 0.63 |
| Economic inequality | 1.04 | 0.96 | 1.04 | 0.96 |
| Mean income per capita | 1.13 | 0.88 | 1.13 | 0.88 |

Note: WP = Within-person; BP = Between-person

***Diagnostic plots for the final model about affective polarization toward politicians***

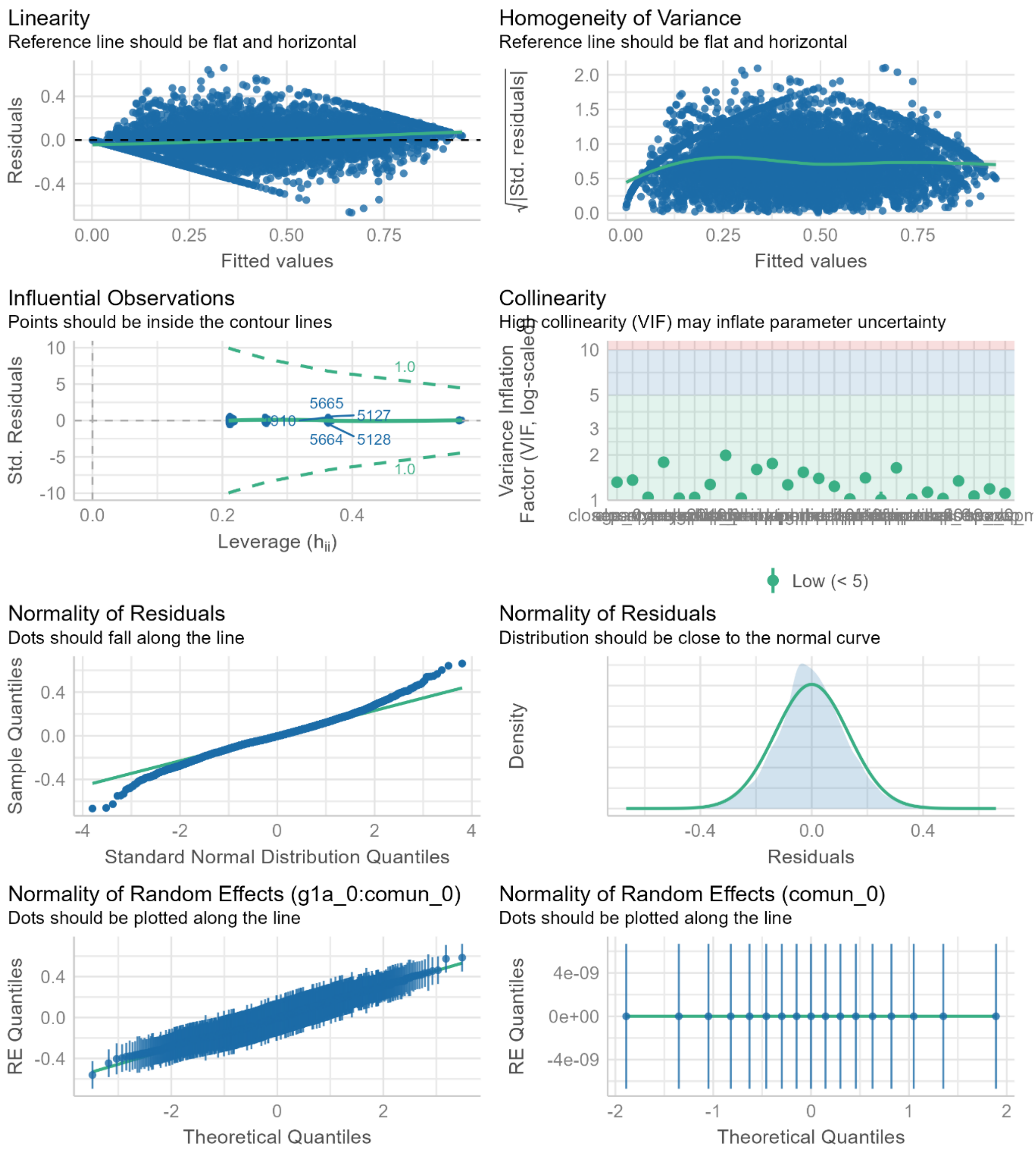

*Diagnostic plots for the final model about affective polarization toward partisans*

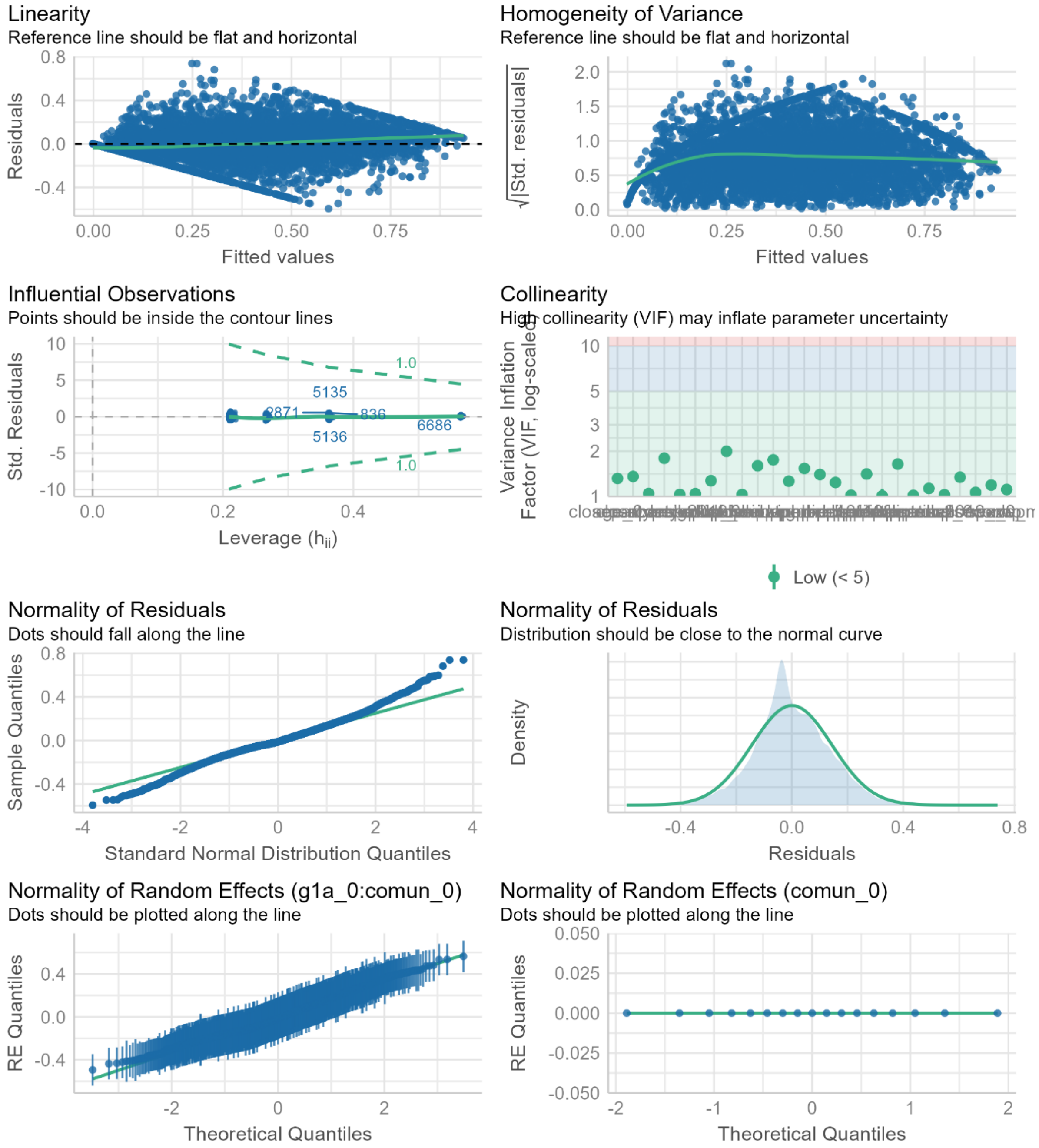

# 6. Exploratory analyses: Interactions tested Diagnostics

We tested the interaction terms between political ideology and predictor variables related to economic threats and identity. We followed a broad test approach for assessing the moderating role of political ideology. This means that we included all the interactions to be tested in the same model, which is recommended when there are no specific hypotheses to test—given the variety of indicators we have—and it allows us to detect effects despite the complementarity among interactions (Harlington & Hayes, 2017[1]). In other words, every interaction term is tested while accounting for other potential interactions that may influence the outcome variable. For instance, satisfaction with the Spanish economy interacts strongly with political ideology, even though political ideology also conditions the role of regional and political identity. In Table S6, we show exclusively all the interaction terms for each outcome variable:

Table S6. Interaction terms added to the multilevel regression models reported in the manuscript

| *Outcome variable* | *Interaction term* | *b* | *SE* | *t* | *df* | *p* |
|---|---|---|---|---|---|---|
| AP Politicians | | | | | | |
| | Political ideology (BP)*Personal economic hardship (WP) | -0.004 | 0.002 | -1.758 | 4930.21 | .079 |
| | Political ideology (BP)*Personal economic concern (WP) | -0.002 | 0.002 | -0.868 | 4991.41 | .385 |
| | Political ideology (BP)*Satisfaction spanish economy (WP) | **-0.002** | **0.001** | **-3.275** | **4916.25** | **.001** |
| | Political ideology (BP)*Perceived unfairness wealth distribution (WP) | -0.001 | 0.001 | -1.453 | 4945.94 | .146 |
| | Political ideology (BP)*Political identity (WP) | **-0.007** | **0.003** | **-2.089** | **4921.87** | **.037** |
| | Political ideology (BP)*Personal economic hardship (BP) | -0.001 | 0.003 | -0.337 | 2070.29 | .736 |
| | Political ideology (BP)*Personal economic concern (BP) | **-0.007** | **0.003** | **-2.276** | **2067.18** | **.023** |
| | Political ideology (BP)*Satisfaction spanish economy (BP) | **-0.008** | **0.001** | **-7.808** | **2068.21** | **< .001** |
| | Political ideology (BP)*Perceived unfairness of the wealth distribution (BP) | **-0.009** | **0.001** | **-9.876** | **2025.04** | **<.001** |
| | Political ideology (BP)*Political identity (BP) | 0.005 | 0.005 | 1.143 | 2056.09 | .253 |

[1] Darlington, R. B., & Hayes, A. F. (2017). Regression Analysis and Linear Models: Concepts, Applications, and Implementation. New York: The Guilford Press.

Table S6. Interaction terms added to the multilevel regression models reported in the manuscript

| *Outcome variable* | *Interaction term* | *b* | *SE* | *t* | *df* | *p* |
|---|---|---|---|---|---|---|
| | Political ideology (BP)*Regional identity (BP) | **0.002** | **0.001** | **2.159** | **2054.5** | **.031** |
| | Political ideology (BP)*National identity (BP) | 0.001 | 0.001 | 1.885 | 2024.72 | .060 |
| | Political ideology (BP)*European identity (BP) | 0.001 | 0.001 | 1.129 | 2031.68 | .259 |
| AP partisans | | | | | | |
| | Political ideology (BP)*Personal economic hardship (WP) | -0.001 | 0.002 | -0.488 | 4966.91 | .626 |
| | Political ideology (BP)*Personal economic concern (WP) | 0.002 | 0.002 | 0.614 | 5007.49 | .539 |
| | Political ideology (BP)*Satisfaction spanish economy (WP) | **-0.002** | **0.001** | **-2.644** | **4935.96** | **.008** |
| | Political ideology (BP)*Perceived unfairness wealth distribution (WP) | -0.001 | 0.001 | -1.298 | 4961.74 | .194 |
| | Political ideology (BP)*Political identity (WP) | **-0.010** | **0.003** | **-2.786** | **4934.52** | **.005** |
| | Political ideology (BP)* Personal economic hardship (BP) | -0.001 | 0.003 | -0.373 | 2065.11 | .709 |
| | Political ideology (BP)*Personal economic concern (BP) | 0.000 | 0.003 | 0.129 | 2061.5 | .897 |
| | Political ideology (BP)*Satisfaction spanish economy (BP) | **-0.008** | **0.001** | **-7.154** | **2064.43** | **<.001** |
| | Political ideology (BP)*Perceived unfairness of the wealth distribution (BP) | **-0.011** | **0.001** | **-10.932** | **2024.12** | **<.001** |
| | Political ideology (BP)*Political identity (BP) | **0.015** | **0.005** | **2.933** | **2053.04** | **.003** |
| | Political ideology (BP)*Regional identity (BP) | **0.002** | **0.001** | **2.555** | **2050.69** | **.011** |
| | Political ideology (BP)*National identity (BP) | 0.001 | 0.001 | 1.848 | 2018.16 | .065 |
| | Political ideology (BP)*European identity (BP) | 0.001 | 0.001 | 1.183 | 2028.98 | .237 |

Note: AP = Affective Polarization; WP = Within-person; BP = Between-person

## 7. Exploratory analyses: Simple slopes analyses for interactions non-consistent across measures of affective polarization

Exploratory analyses showed that political ideology moderated the association between regional identity and affective polarization. Among left-wing people (+1SD), the more regional identity, the less affective polarization toward politicians ($b_{Politicians}$ = -0.005, $p$ = .046); while, among right-wing people, regional identity was not associated with affective polarization toward politicians ($b_{politicians}$= 0.002, $p$ = .389). Although the pattern was similar for affective polarization toward partisans, simple slope analyses showed that the association between regional identity and affective polarization was non-statistically significant at +/- 1 SD from the mean: For left-wing, $b_{Partisans}$ = -0.005, $p$ = .084; and right-wing, $b_{Partisans}$ = .005, $p$ = .101. However, this relationship becomes statistically significant if we test at 2 SD above and below the mean: For far left-wing, $b_{Partisans}$ = -0.008, $p$ = .024; and for far right-wing, $b_{Partisans}$ = 0.009, $p$ = .030 (see Figure S4).

***Figure S4.*** *Simple slopes analyses of the association between Regional Identity and affective polarization toward politicians (Panel A) and toward partisans (Panel B), at different levels of political ideology.*

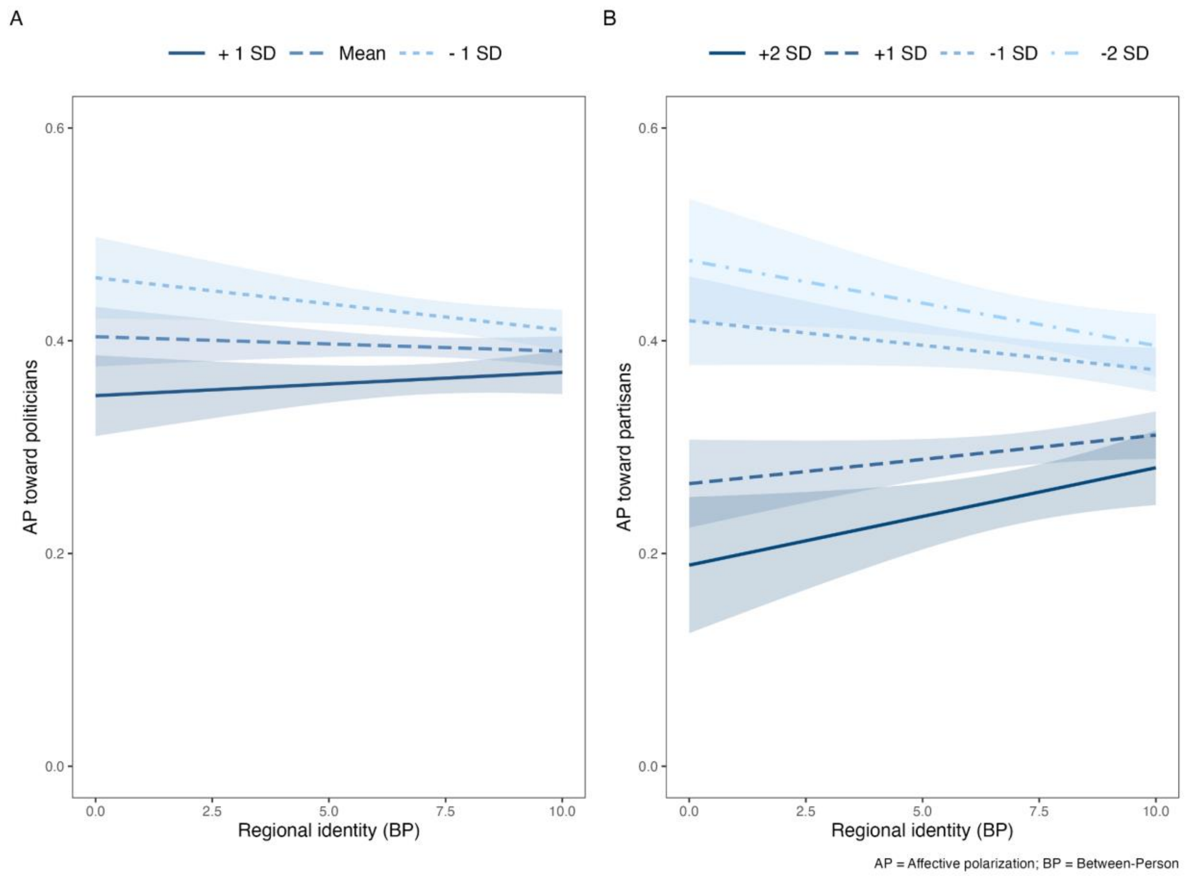

Furthermore, the relationship between affective polarization and personal economic concerns was non-statistically significant for people 1 SD above or below the mean (-1SD, *b* = 0.019, *p* = .070; +1SD, *b* = -0.012, *p* = .216). However, this relationship was positive and statistically significant for people on the far-left side of the political spectrum (-2SD, *b* = 0.031, *p* = .034). Yet, it was negative and marginally significant for people placed on the far right-wing people (+2SD, b = -0.028, *p* = .069) (see Fig. S5).

***Figure S5.*** *Simple slopes analyses of the association between personal economic concerns (between-person) and affective polarization toward politicians at different levels of political ideology.*

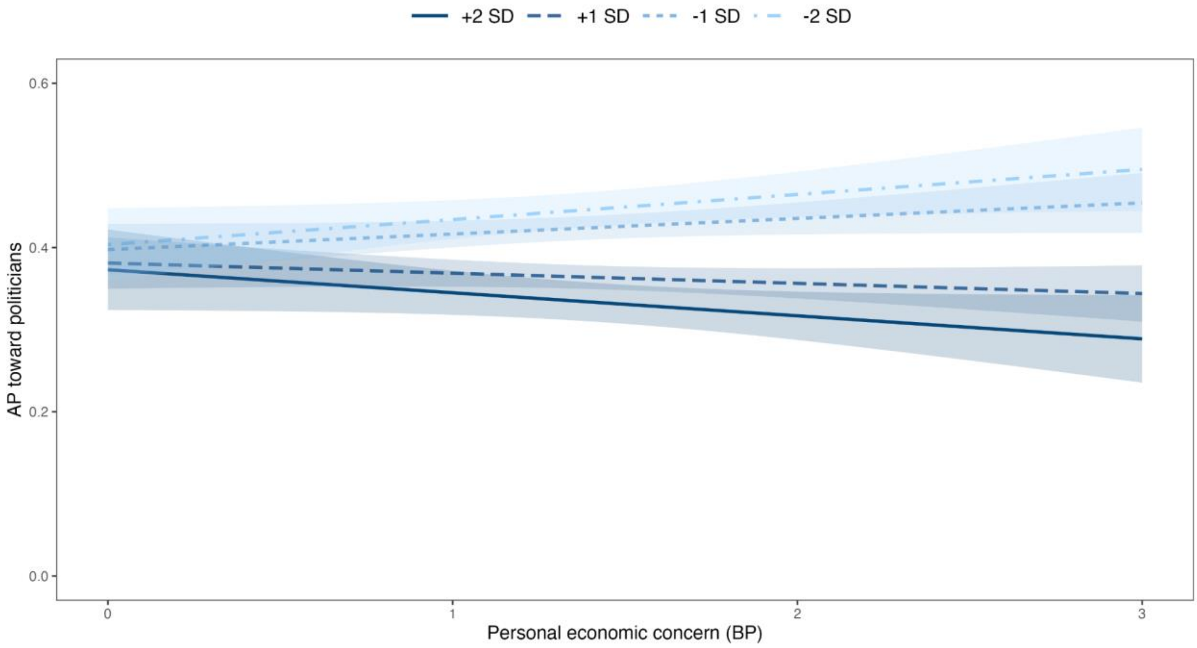

## 8. Robustness checks: Testing personal economic threats separately

**Table S7. Multilevel regression analyses using personal economic threat indicators separately**

| | **Affective polarization toward politicians** | | | **Affective polarization toward partisans** | | |
|---|---|---|---|---|---|---|
| | *Full model* | *Only Personal economic hardship* | *Only Personal economic concern* | *Full model* | *Only Personal economic hardship* | *Only Personal economic concern* |
| *Predictors* | *β (SE)* | *β (SE)* | *β (SE)* | *β (SE)* | *β (SE)* | *β (SE)* |
| (Intercept) | -0.000 (0.016) | -0.000 (0.016) | -0.000 (0.016) | -0.001 (0.016) | -0.001 (0.016) | -0.001 (0.016) |
| Wave (months) | 0.009 (0.007) | 0.009 (0.007) | 0.009 (0.007) | 0.009 (0.007) | 0.009 (0.007) | 0.010 (0.007) |
| Age (years) | 0.089 *** (0.018) | 0.090 *** (0.018) | 0.084 *** (0.018) | 0.032 (0.019) | 0.035 (0.018) | 0.028 (0.018) |
| Sex (Female) | -0.020 (0.017) | -0.020 (0.017) | -0.018 (0.017) | -0.004 (0.018) | -0.005 (0.018) | -0.003 (0.018) |
| Education | -0.018 (0.018) | -0.018 (0.018) | -0.016 (0.018) | -0.032 (0.018) | -0.031 (0.018) | -0.030 (0.018) |
| Income | 0.046 * (0.019) | 0.047 * (0.019) | 0.056 ** (0.018) | 0.020 (0.019) | 0.021 (0.019) | 0.026 (0.018) |
| Political ideology (BP) | -0.094 *** (0.020) | -0.094 *** (0.020) | -0.093 *** (0.020) | -0.117 *** (0.020) | -0.117 *** (0.020) | -0.116 *** (0.020) |
| Residence (rural) | 0.015 (0.016) | 0.015 (0.016) | 0.015 (0.016) | 0.012 (0.016) | 0.012 (0.016) | 0.012 (0.016) |
| Media consumption (BP) | -0.004 (0.018) | -0.004 (0.018) | -0.004 (0.018) | 0.044 * (0.018) | 0.043 * (0.018) | 0.044 * (0.018) |
| Media consumption (WP) | 0.021 ** (0.007) | 0.021 ** (0.007) | 0.021 ** (0.007) | 0.009 (0.007) | 0.009 (0.007) | 0.009 (0.007) |
| Political interest (BP) | 0.162 *** (0.020) | 0.162 *** (0.020) | 0.162 *** (0.020) | 0.114 *** (0.021) | 0.114 *** (0.021) | 0.114 *** (0.021) |
| Political interest (WP) | 0.021 ** (0.007) | 0.021 ** (0.007) | 0.021 ** (0.007) | 0.027 *** (0.007) | 0.027 *** (0.007) | 0.027 *** (0.007) |
| Personal economic hardship (WP) | 0.008 (0.007) | 0.008 (0.007) | | **0.017 * (0.007)** | **0.018 * (0.007)** | |
| Personal economic concern (WP) | -0.001 (0.007) | | 0.001 (0.007) | 0.004 (0.007) | | 0.006 (0.007) |
| Satisfaction with Economy (WP) | 0.003 (0.007) | 0.003 (0.007) | 0.003 (0.007) | -0.003 (0.007) | -0.003 (0.007) | -0.004 (0.007) |
| Perceived unfairness of wealth redistribution | 0.021 ** (0.007) | 0.021 ** (0.007) | 0.021 ** (0.007) | 0.015 * (0.007) | 0.015 * (0.007) | 0.015 * (0.007) |

**Table S7. Multilevel regression analyses using personal economic threat indicators separately**

| | | | | | | |
|---|---|---|---|---|---|---|
| Partisan identity | 0.033 *** (0.007) | 0.033 *** (0.007) | 0.033 *** (0.007) | 0.040 *** (0.007) | 0.041 *** (0.007) | 0.040 *** (0.007) |
| Personal economic hardship (BP) | -0.037 (0.022) | **-0.040 * (0.019)** | | -0.025 (0.023) | -0.034 (0.020) | |
| Personal economic concern (BP) | -0.005 (0.021) | | -0.023 (0.018) | -0.016 (0.022) | | -0.028 (0.019) |
| Satisfaction with Economy (BP) | -0.042 * (0.018) | -0.042 * (0.018) | -0.037 * (0.018) | -0.069 *** (0.019) | -0.068 *** (0.019) | -0.066 *** (0.018) |
| Perceived unfairness of wealth redistribution (BP) | 0.019 (0.019) | 0.019 (0.019) | 0.016 (0.019) | 0.038 * (0.019) | 0.038 * (0.019) | 0.037 (0.019) |
| Partisan identity (BP) | 0.363 *** (0.018) | 0.363 *** (0.018) | 0.363 *** (0.018) | 0.363 *** (0.019) | 0.363 *** (0.019) | 0.362 *** (0.019) |
| Regional identity (BP) | -0.003 (0.018) | -0.003 (0.018) | -0.003 (0.018) | 0.009 (0.018) | 0.009 (0.018) | 0.009 (0.018) |
| National identity (BP) | 0.117 *** (0.021) | 0.116 *** (0.021) | 0.116 *** (0.021) | 0.057 ** (0.021) | 0.055 ** (0.021) | 0.056 ** (0.021) |
| European identity (BP) | -0.025 (0.020) | -0.025 (0.020) | -0.024 (0.020) | -0.035 (0.020) | -0.035 (0.020) | -0.035 (0.020) |
| Economic inequality (region) | -0.011 (0.016) | -0.011 (0.016) | -0.012 (0.016) | -0.024 (0.016) | -0.024 (0.016) | -0.025 (0.016) |
| Income (region) | 0.018 (0.017) | 0.018 (0.017) | 0.018 (0.017) | 0.006 (0.017) | 0.007 (0.017) | 0.007 (0.017) |
| **Random Effects** | | | | | | |
| $\sigma^2$ | 0.023 | 0.023 | 0.023 | 0.027 | 0.027 | 0.027 |
| $\tau_{00}$ | 0.029 | 0.029 | 0.029 | 0.035 | 0.035 | 0.035 |
| | $0.000_{comun\_0}$ | $0.000_{comun\_0}$ | $0.000_{comun\_0}$ | $0.000_{comun\_0}$ | $0.000_{comun\_0}$ | $0.000_{comun\_0}$ |
| N | $2051_{g1a\_0}$ | $2051_{g1a\_0}$ | $2051_{g1a\_0}$ | $2047_{g1a\_0}$ | $2047_{g1a\_0}$ | $2047_{g1a\_0}$ |
| | $17_{comun\_0}$ | $17_{comun\_0}$ | $17_{comun\_0}$ | $17_{comun\_0}$ | $17_{comun\_0}$ | $17_{comun\_0}$ |
| Observations | 6933 | 6933 | 6933 | 6944 | 6944 | 6944 |
| Marginal $R^2$ | 0.449 | 0.449 | 0.448 | 0.412 | 0.411 | 0.411 |

* *p<.05* ** *p<.01* *** *p<.001*

# 9. Robustness checks: Testing identities separately

**Table S7. Multilevel regression analyses using personal economic threat indicators separately**

| | *Affective polarization toward politicians* | | | | | *Affective polarization toward partisans* | | | | |
|---|---|---|---|---|---|---|---|---|---|---|
| | *Full model* | *Only regional identity* | *Only National identity* | *Only European identity* | *Only Partisan identity* | *Full model* | *Only regional identity* | *Only National identity* | *Only European identity* | *Only Partisan identity* |
| *Predictors* | *β (SE)* | *β (SE)* | *β (SE)* | *β (SE)* | *β (SE)* | *β (SE)* | *β (SE)* | *β (SE)* | *β (SE)* | *β (SE)* |
| Partisan identity (WP) | 0.033 *** (0.007) | | | | 0.033 *** (0.007) | 0.040 *** (0.007) | | | | 0.041 *** (0.007) |
| Partisan identity (BP) | 0.363 *** (0.018) | | | | 0.366 *** (0.018) | 0.363 *** (0.019) | | | | 0.363 *** (0.019) |
| Regional identity (BP) | -0.003 (0.018) | 0.059 *** (0.018) | | | | 0.009 (0.018) | 0.051 ** (0.018) | | | |
| National identity (BP) | 0.117 *** (0.021) | | 0.116 *** (0.020) | | | 0.057 ** (0.021) | | 0.056 ** (0.021) | | |
| European identity (BP) | -0.025 (0.020) | | | 0.045 * (0.019) | | -0.035 (0.020) | | | 0.014 (0.019) | |
| Covariates | Yes | Yes | Yes | Yes | Yes | Yes | Yes | Yes | Yes | Yes |
| Focal predictors | Yes | Yes | Yes | Yes | Yes | Yes | Yes | Yes | Yes | Yes |
| $\sigma^2$ | 0.023 | 0.023 | 0.023 | 0.023 | 0.023 | 0.027 | 0.027 | 0.027 | 0.027 | 0.027 |
| $\tau_{00}$ | 0.029 | 0.037 | 0.036 | 0.037 | 0.030 | 0.035 | 0.043 | 0.043 | 0.043 | 0.035 |
| ICC | | 0.615 | | 0.616 | 0.566 | | 0.611 | | | |

*Note: * p<.05 ** p<.01 *** p<.001;* The effects of covariates and focal predictors did not vary by including identity variables separately, which could be seen in the final models reported in the manuscript. We only focused on identity variables to examine whether each explains an independent portion of the variance or whether they become non-significant when other identities are present. The gray-shaded text indicates variables that change their statistical significance when tested independently.
